# Developmental capacity is unevenly distributed among single blastomeres of 2-cell and 4-cell stage mouse embryos

**DOI:** 10.1038/s41598-021-00834-1

**Published:** 2021-11-02

**Authors:** Katarzyna Krawczyk, Ewa Kosyl, Karolina Częścik-Łysyszyn, Tomasz Wyszomirski, Marek Maleszewski

**Affiliations:** 1 Department of Embryology, Institute of Developmental Biology and Biomedical Sciences, Faculty of Biology, University of Warsaw, Miecznikowa 1, 02-096 Warsaw, Poland; 2 Department of Ecology and Environmental Protection, Institute of Environmental Biology, Faculty of Biology, University of Warsaw, Żwirki i Wigury 101, 02-089 Warsaw, Poland

**Keywords:** Cell biology, Developmental biology

## Abstract

During preimplantation development, mammalian embryo cells (blastomeres) cleave, gradually losing their potencies and differentiating into three primary cell lineages: epiblast (EPI), trophectoderm (TE), and primitive endoderm (PE). The exact moment at which cells begin to vary in their potency for multilineage differentiation still remains unknown. We sought to answer the question of whether single cells isolated from 2- and 4-cell embryos differ in their ability to generate the progenitors and cells of blastocyst lineages. We revealed that twins were often able to develop into blastocysts containing inner cell masses (ICMs) with PE and EPI cells. Despite their capacity to create a blastocyst, the twins differed in their ability to produce EPI, PE, and TE cell lineages. In contrast, quadruplets rarely formed normal blastocysts, but instead developed into blastocysts with ICMs composed of only one cell lineage or completely devoid of an ICM altogether. We also showed that quadruplets have unequal capacities to differentiate into TE, PE, and EPI lineages. These findings could explain the difficulty of creating monozygotic twins and quadruplets from 2- and 4-cell stage mouse embryos.

## Introduction

Totipotency in its strict sense is defined as the ability of a single cell to produce a fertile adult individual^1,2^. Mammalian zygotes, for example, can differentiate into all types of embryonic cells and transient extraembryonic structures, such as the placenta and fetal membranes. Moreover, a single blastomere isolated from a 2-cell mouse embryo can also develop into a blastocyst and then into offspring^3^; however, attempts to experimentally obtain monozygotic twins in this species have rarely been successful^4–9^. This observation does not contradict the totipotency of 2-cell stage mouse blastomeres, but rather supports the idea that sister blastomeres are not equally totipotent^8,9^. In the mouse, starting at the 4-cell stage, single isolated blastomeres are not able to produce a functional blastocyst, which is most likely due to the insufficient number of cells at the time of implantation^10–18^.

Despite the fact that mouse blastomeres are morphologically indistinguishable up to the 8-cell stage, there are many lines of evidence suggesting that they not only differ in mRNA composition but also exhibit remarkably different expression of protein-coding genes, including genes that code for signaling molecules, transcription factors, and epigenetic modifiers^19–22^. It has been suggested that interblastomere differences play a central role in cell fate decision, namely, the specification of the inner cell mass (ICM) and trophectoderm (TE). Two long noncoding RNAs, *LincGET* and *Neat1*, are differently distributed among sister blastomeres of mouse embryos already at the 2-cell stage^23,24^. These transcripts interact with coactivator-associated arginine methyltransferase 1 (CARM1) and promote the commitment of a pluripotent ICM^23,24^. At the 4-cell stage, the heterogeneous activity of CARM1, interacting with PR domain-containing protein 14 (PRDM14), results in differential methylation of arginine 26 in histone H3 (H3R26me2)^25,26^. Hence, blastomeres with increased levels of histone H3R26me2 show higher expression of the pluripotency genes responsible for establishing the ICM lineage, such as *Nanog* and *Sox2*^25^. It has been demonstrated that *Sox21*, which is a target of SOX2, is also asymmetrically expressed between blastomeres in 4-cell mouse embryos^27^. Consistently, cells with lower *Sox21* expression upregulate the TE-specific transcription factor CDX2 and contribute to the extraembryonic lineage. This finding is consistent with a previous report showing that distribution of *Cdx2* mRNA is heterogeneous among mouse blastomeres at the 8-cell stage^28^. Cells with elevated *Cdx2* levels tend to become the future TE, while those with minimal *Cdx2* expression contribute preferentially to the ICM.

Another factor that can serve as a predictive measure of future lineage segregations in the early mouse embryo is the differential kinetic behaviors of specific transcription factors. Quantitative analyses of DNA-binding dynamics have revealed distinct kinetics of SOX2 and OCT4 at the 4-cell stage, which play a pivotal role in specifying the pluripotent cell lineage^29,30^. Cells exhibiting slower SOX2 and OCT4 kinetics, associated with their greater retention in the nucleus and increased DNA-binding affinities, are more likely to give rise to the ICM lineage. By contrast, cells with faster kinetics of these factors, manifested by lower nuclear retention rates and decreased accessibility to DNA binding sites, contribute mostly to the TE. These results together suggest that cells as early as at the 2- and 4-cell stages can already exhibit differences in fate-determining gene expression patterns and in the activity of specific cell-fate regulators, leading to unequal developmental fate and potential.

Many studies have traced the cell fate of labeled blastomeres of 2-and 4-cell mouse embryos. Although some have claimed that the first two blastomeres display developmental biases in contributing to either the ICM or TE lineages^8,31,32^, others have failed to confirm such a relationship^33–39^. There are several lines of evidence demonstrating that the progeny of some blastomeres labeled at the 4-cell stage can contribute only to embryonic or extraembryonic tissue^34,39,40^. In accordance with these results, it has been reported that the 4-cell embryo contains developmentally nonequivalent blastomeres, which differ in their individual abilities to produce chimeric embryos^41^. Based on the findings mentioned above, one may wonder whether sister blastomeres in the intact embryo behave in the same manner as blastomeres that were separated from each other. To address this question, the cell fate determination of isolated blastomeres has been investigated using molecular markers for cell subpopulations of the resultant blastocysts^8,42^. The cell lineages of the blastocyst arise from two differentiation events, TE *vs.* ICM, followed by epiblast (EPI) *vs.* primitive endoderm (PE) formation within the ICM^43^. The pluripotent EPI provides the foundation for the future body of the fetus, while the extraembryonic tissues, the TE and PE, are essential for the development of the placenta and yolk sac, respectively. So far, it has been demonstrated that monozygotic twin blastocysts derived from bisected 2-cell stage mouse embryos differ in their relative numbers of ICM and TE cells^42^. Recently, more careful inspection of twins has revealed functional differences in the formation of their EPI^8^. In this context, whether the heterogeneity in the individual developmental properties between sister blastomeres manifests more clearly at the late blastocyst stage remains an open question. Finally, it remains unknown whether isolated 4-cell stage sister blastomeres are equivalent in terms of developmental potential.

In this study, we aimed to examine whether single cells isolated from 2- and 4-cell embryos differ in their ability to generate the progenitors and cells of blastocyst lineages (TE, EPI, and PE). The pairs (twins) and sets of four single blastomeres (quadruplets) were cultured to the early or late blastocyst stage, then the cell lineage allocation was analyzed. We revealed that twins were often able to develop into blastocysts containing ICMs with PE and EPI cells. Yet despite their capacity to create a blastocyst, the twins differed in their ability to produce EPI, PE, and TE cell lineages when they reached the late blastocyst stage. In contrast, quadruplets rarely formed normal blastocysts and instead developed into blastocysts with ICMs composed of only one cell lineage or completely devoid of an ICM altogether. This finding indicates that their potency is limited compared to 2-cell stage blastomeres. We also showed that quadruplets feature unequal capacities to differentiate into TE, PE, and EPI lineages.

## Results

### Preimplantation development of twin embryos obtained by disaggregation of 2-cell embryos

#### The frequency of development to the blastocyst stage

**Figure 1:**
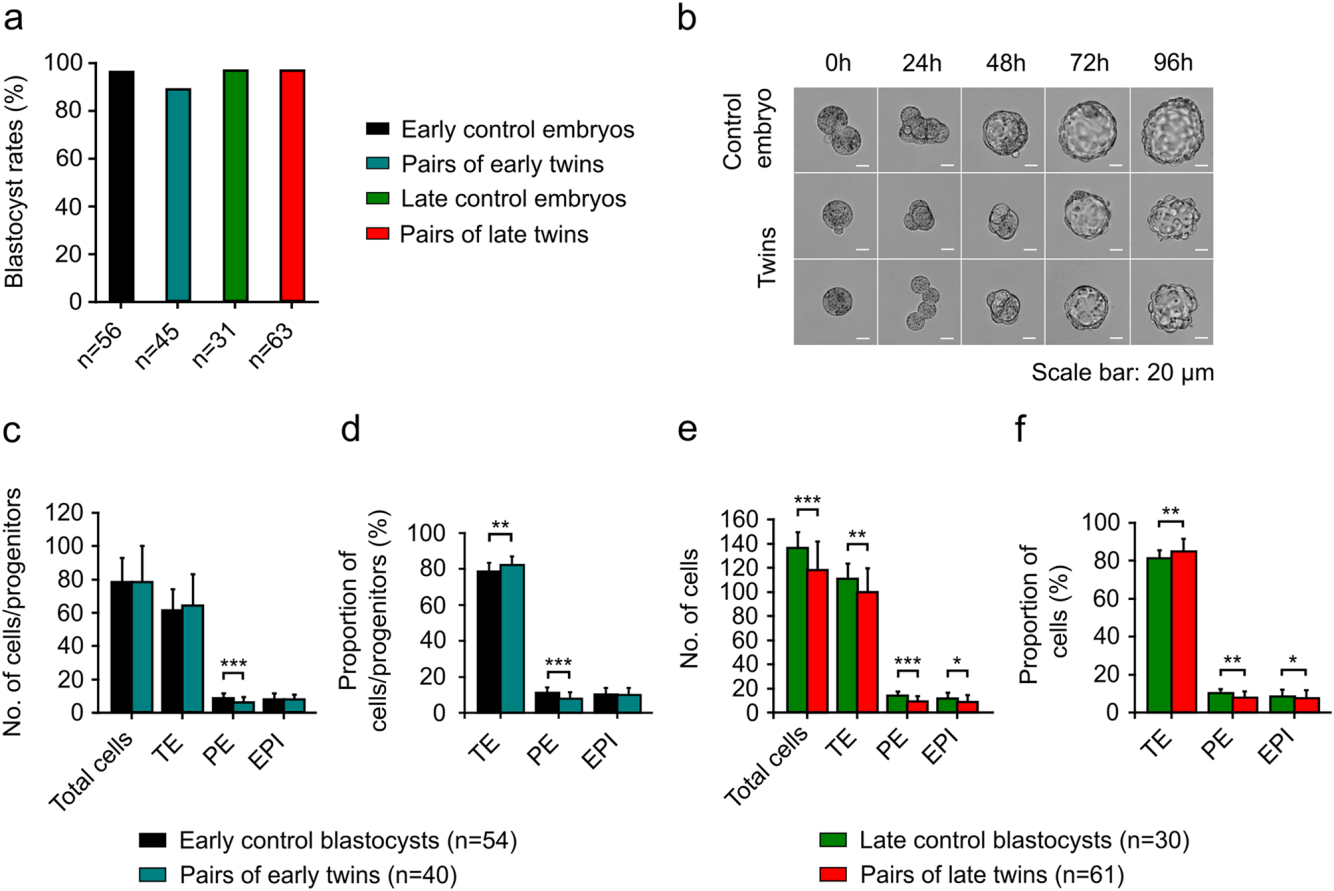
Cell lineage allocation in monozygotic twin blastocysts and controls. (**a**) Blastocyst development from 2-cell stage single blastomeres after 72 (early variation) or 96 (late variation) hours of in vitro culture. The data were obtained from 7 experiments. The results were compared using Chi-squared test. (**b**) Representative brightfield images showing embryos developed from 2-cell stage single blastomeres after 24, 48, 72, and 96 h of in vitro culture. Scale bar: 20 µm. (**c**) Quantitative analysis of the total number of cells, the number of CDX2-positive cells (TE), as well as the numbers of SOX2-positive (EPI) and SOX17-positive (PE) progenitors in pairs of early twin blastocysts. (**d**) The percentage of TE cells, as well as EPI and PE progenitors in pairs of early twin blastocysts. (**e**) Quantification of the total number of cells, as well as the numbers of TE, EPI, and PE cells in pairs of late twin blastocysts. (**f**) Percentages of TE, EPI, and PE cells relative to the total number of cells in pairs of late twin blastocysts. For (**c**, **d**, **e**, and **f**), normality of the data was verified with the Kolmogorov–Smirnov test, and Student’s t-test was used for calculations. *p < 0.05, **p < 0.01, ***p < 0.001. Error bars indicate standard deviations.

In the first (early) variation of the experiment, single blastomeres of 2-cell embryos and control embryos were cultured in vitro for 72 h. As a result of 2-cell embryo disaggregation, we obtained 45 pairs of blastomeres (in 7 experiments), of which 40 (88.9%) developed to the early blastocyst stage (Fig. 1a, b). Unmanipulated control embryos at the 2-cell stage (n = 56) provided a basis for comparison. After 72 h of culture, 54 out of 56 (96.4%) control embryos reached the early blastocyst stage (Fig. 1a, b). Both the control and experimental embryos progressed to this stage with similar frequency. In the second (late) variation of the experiment, single blastomeres of 2-cell embryos and control embryos were cultured in vitro for 96 h. As a result of disaggregation, we obtained 63 pairs of blastomeres (in 7 experiments), of which 61 (96.8%) formed late blastocysts (Fig. 1a, b). The rate of cell lysis caused by the disaggregation with a thin glass needle yielded 49% in both experimental variations. In 31 unmanipulated control 2-cell embryos, almost all (30 out of 31; 96.8%) developed to the late blastocyst stage (Fig. 1a, b). Thus, monozygotic twins and control embryos formed late blastocysts at similar rates.

#### Cell lineage allocation in monozygotic twin blastocysts and controls

We analyzed the cell lineage allocation in pairs of blastocysts (twins) from the early variation of the experiment (72 h of culture) and compared it with the corresponding control blastocysts. Confocal images of immunofluorescent-labeled blastocysts were used to determine the total number of cells, as well as the number and proportion of SOX2-positive (EPI), SOX17-positive (PE), and CDX2-positive (TE) cells. While we did observe cells co-expressing SOX2 and SOX17 markers, for the purposes of the statistical analysis, we categorized them as SOX2- or SOX17-positive cells depending on immunofluorescence signal strength. The data for the experimental group were analyzed as the sum of the respective cells for twin blastocysts developed from a single embryo, and Student’s t-tests were used for all analyses.

We revealed that twin blastocysts in the early variation had a similar total number of cells as unmanipulated controls (78.3 ± 22.0 *vs.* 78.3 ± 14.5) and did not differ in the numbers of TE cells or EPI progenitors (Fig. 1c; Supplementary Tables S1, S2). The mean numbers of TE cells in twins and controls were 64.3 ± 18.9 and 61.6 ± 12.5, respectively. In twins, the EPI contained on average 7.8 ± 3.2 progenitors, which was similar to the cell number observed in EPI in control blastocysts (8.0 ± 3.5). The only difference observed between the groups was associated with the PE lineage (Fig. 1c; Supplementary Tables S1, S2). The mean number of PE progenitors was significantly lower in twins (6.2 ± 3.2) compared to control embryos (8.7 ± 2.8; *p* = 0.0001).

We also analyzed the proportions of cells contributing to cell lineages in twin and control blastocysts (Fig. 1d; Supplementary Tables S1, S2). In the early twin blastocysts, the proportion of TE cells was higher than in control blastocysts (82.1% ± 4.8% and 78.7% ± 4.8%; *p* = 0.005). Twin embryos also had a lower proportion of PE progenitors than controls (7.9% ± 3.5% and 11.0% ± 3.3%; *p* < 0.0001). The fraction of EPI progenitors was the same in both groups (10.0% ± 4.0% for each group).

Next, we analyzed the number of cells of TE, PE, and EPI lineages in twin blastocysts and control blastocysts at 96 h of culture (Fig. 1e; Supplementary Tables S3, S4). The total number of cells in pairs of late twin blastocysts was lower than that in control blastocysts (117.9 ± 23.6 and 136.4 ± 12.9; *p* = 0.0001). We also noted that all analyzed cell lineages in twins were composed of a smaller number of cells compared to controls. The number of TE cells averaged 99.9 ± 19.8 in late twins and 110.7 ± 12.5 in controls (*p* = 0.008). The ICMs of late twins contained on average 9.1 ± 4.3 PE cells (*p* = 0.0001) and 8.9 ± 5.5 EPI cells (*p* = 0.025), whereas the ICMs of controls averaged 14.1 ± 3.2 PE cells and 11.6 ± 4.7 EPI cells.

Then we investigated whether late twins and control blastocysts differ in the proportions of cells in each lineage (Fig. 1f; Supplementary Tables S3, S4). We found that in late twins, the proportion of TE cells was higher than in control blastocysts (84.8% ± 6.8% and 81.2% ± 4.4%; *p* = 0.005). Moreover, late twins were characterized by lower proportions of PE and EPI cells compared to controls. Twin blastocysts had an average of 7.7% ± 3.3% PE cells (*p* = 0.001) and 7.5% ± 4.2% EPI cells (*p* = 0.018) in the ICMs. In controls, the ICMs contained on average 10.3% ± 2.2% PE cells and 8.5% ± 3.4% EPI cells.

Among the twin blastocysts containing both cell lineages in the ICM, we most often observed embryos with sorted signals for SOX2 and SOX17 markers (90 of 104 twin blastocysts, 86.5%). Less commonly, we observed blastocysts in which ICM cells were only partly segregated (with at least one EPI cell next to the PE cells) due to an insufficient number of PE cells to cover the ICM surface (8 of 104 twin blastocysts, 7.7%). The rarest were embryos in which EPI and PE cells were not segregated into appropriate positions (6 of 104 twin blastocysts, 5.8%). In the control group, all embryos were composed of EPI and PE cells segregated in ICMs.

Taken together, our results demonstrate that the dissimilarities in the number and proportion of cells of individual cell lineages between monozygotic twin blastocysts and controls deepen over the course of development and are more pronounced at the late blastocyst stage. We found that the early twins and controls were similar in terms of the total numbers of cells, the number of TE cells, and the number and proportion of EPI progenitors. In contrast, late twins and controls varied not only in the total numbers of cells but also in the numbers and proportions of cells forming all three cell lineages of the blastocyst (TE, PE, and EPI).

#### Characteristics of monozygotic twin blastocysts developed from one 2-cell embryo

**Figure 2:**
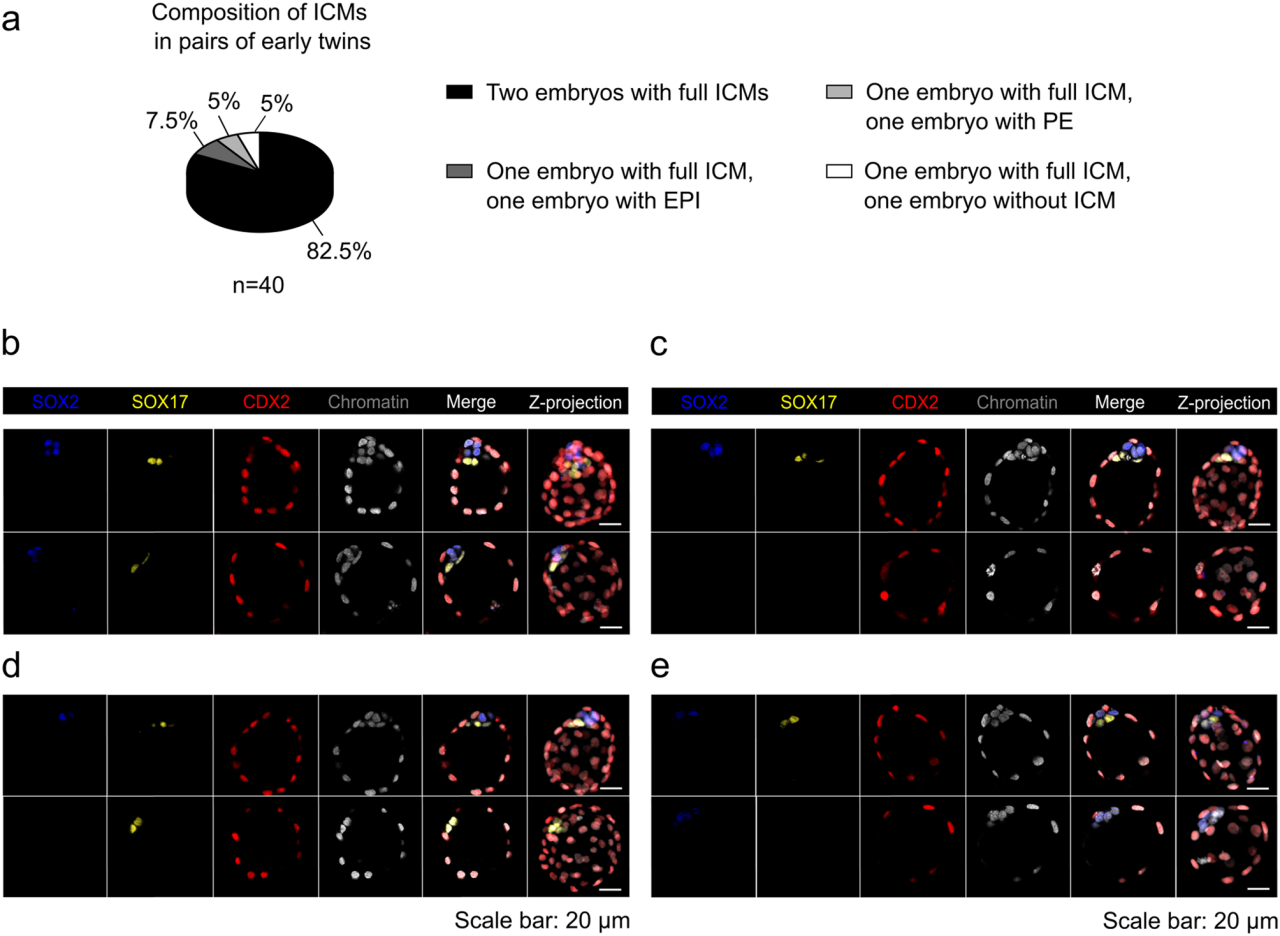
Potency of pairs of early twin blastocysts to generate inner cells. (**a**) Composition of ICMs in pairs of early twin blastocysts. (**b**, **c**, **d**, and **e**) Immunodetection of SOX2 (blue), SOX17 (yellow), and CDX2 (red) in representative twin blastocysts. Chromatin (white) was labeled with chromomycin A_3_. The last panel corresponds to the average intensity projections of Z-stack images. Scale bar: 20 µm. A pair in which both embryos had ICMs consisting of EPI and PE progenitors is shown in (**b**). A pair in which one embryo created a complete ICM and the other developed into a trophoblastic vesicle is presented in (**c**). The pairs in which one embryo created a complete ICM and the other only PE or EPI progenitors are shown in (**d**) and (**e**).

Next, we compared the twin blastocysts developed from the two blastomeres of the 2-cell embryo. In the early variation of the experiment, the dominant pairs of twin blastocysts were those in which both embryos formed an ICM consisting of both PE and EPI progenitors (33 of 40 pairs; 82.5%; Fig. 2a, b). Much less frequently, we observed pairs of embryos in which one embryo developed into a trophoblastic vesicle (2 of 40; 5.0%; Fig. 2a, c) or created only PE (2 of 40; 5.0%; Fig. 2a, d) or EPI (3 of 40; 7.5%; Fig. 2a, e) progenitors.

**Figure 3:**
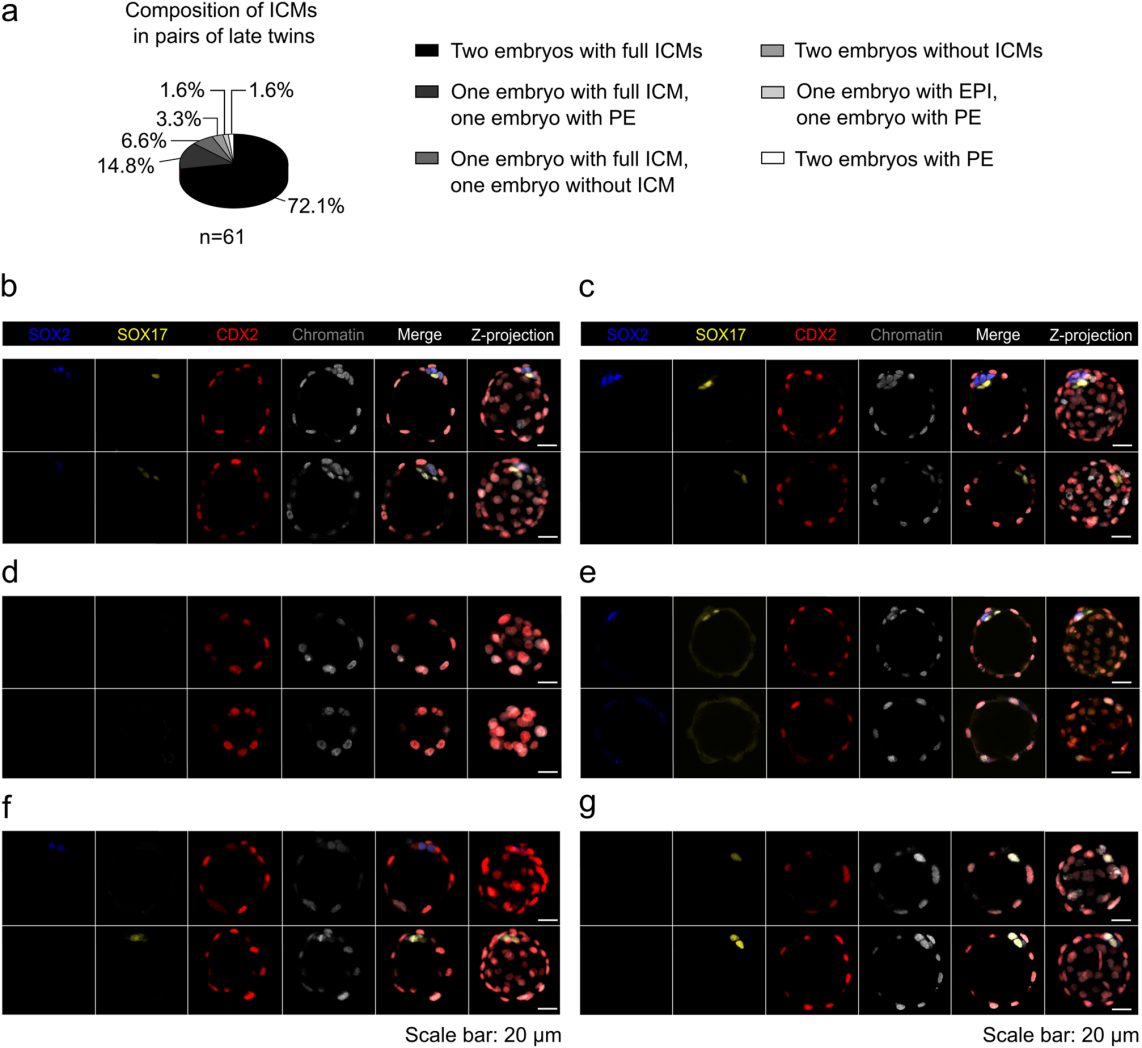
Potency of pairs of late twin blastocysts to generate inner cells. (**a**) Composition of ICMs in pairs of late twin blastocysts. (**b**, **c**, **d**, **e**, **f**, and **g**) SOX2 (blue), SOX17 (yellow), and CDX2 (red) immunolocalization in representative twin blastocysts. Chromatin (white) was labeled with chromomycin A_3_. The last panel corresponds to the average intensity projections of Z-stack images. Scale bar: 20 µm. A pair in which both embryos had ICMs consisting of EPI and PE cells is shown in (**b**). A pair in which one embryo created a complete ICM and the other formed only PE cells is presented in (**c**). A pair in which both embryos formed trophoblastic vesicles is shown in (**d**). A pair in which one embryo created a complete ICM and the other developed into trophoblastic vesicle is presented in (**e**). A pair in which one embryo contained only EPI and the other only PE cells is shown in (**f**). A pair in which both embryos had exclusively PE in their ICMs is presented in (**g**).

Among the pairs of twin blastocysts in the late variation, we most often observed both embryos developing an ICM composed of two lineages: EPI and PE (44 of 61 pairs; 72.1%; Fig. 3a, b). Less commonly, we observed pairs in which one embryo created a complete ICM and the other only PE cells (9 of 61 pairs; 14.8%; Fig. 3a, c). Most rarely, we observed twins in which both embryos (2 of 61 pairs; 3.3%; Fig. 3a, d) or one embryo (4 of 61 pairs; 6.6%; Fig. 3a, e) developed into trophoblastic vesicles or into pairs in which one embryo contained only EPI and the other only PE cells (1 of 61 pairs; 1.6%; Fig. 3a, f). In the remaining one case, both twin embryos had exclusively PE cells in their ICMs (1 of 61 pairs; 1.6%; Fig. 3a, g).

This analysis showed that the vast majority of monozygotic twin blastocysts derived from single blastomeres of 2-cell embryos were capable of generating ICMs consisting of EPI and PE progenitors/cells both in the early and late variations of the experiment. In addition, we found that embryos that reached the early blastocyst stage had slightly greater potential to form complete ICMs than embryos that developed into the late blastocyst stage.

To investigate whether twin blastocysts differ in the number and proportion of cells of each cell lineage, we calculated the absolute value of the difference between the results obtained for each pair of experimental blastocysts. Using Student’s t-test for one mean, we compared the results obtained for twin blastocysts to the reference mean value and the standard deviation of these parameters in randomly matched pairs of blastocysts from the control group. For the purposes of this analysis, we randomly selected 1,465 pairs of blastocysts in the early control group and 1400 pairs of blastocysts in the late control group.

**Figure 4:**
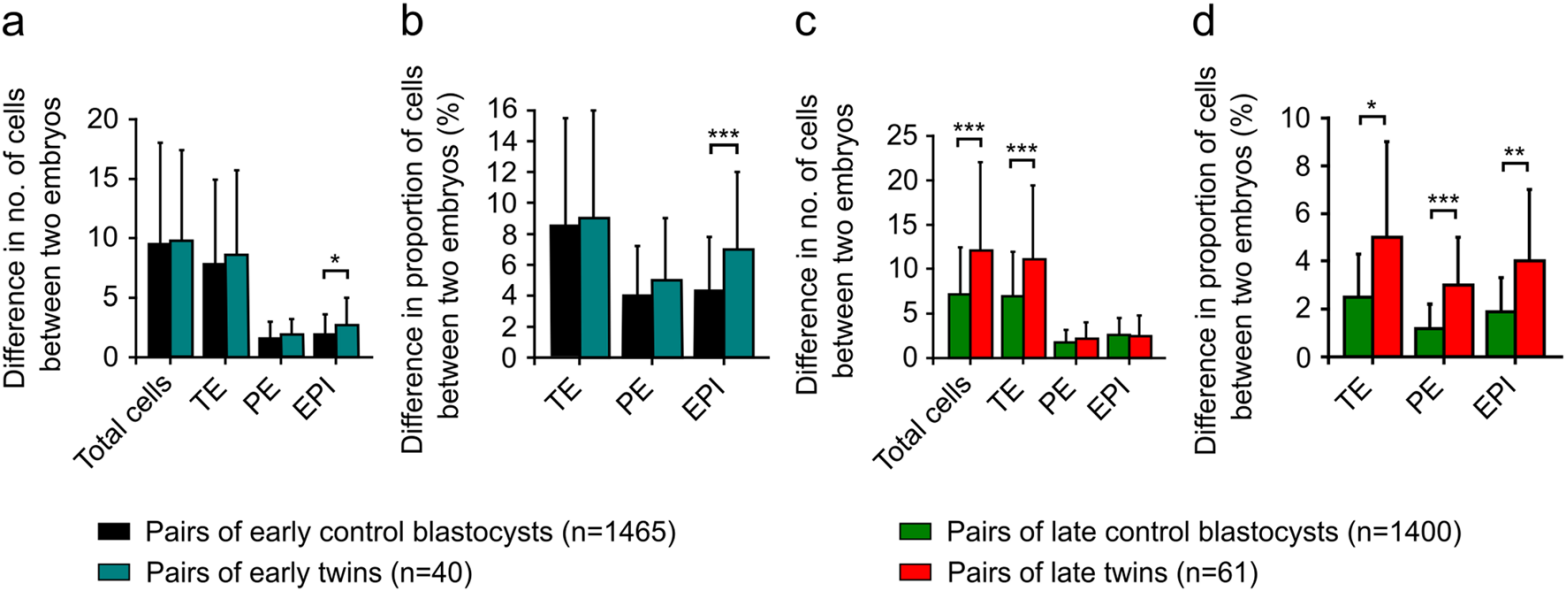
Cell lineage analysis of pairs of early and late twin blastocysts. (**a**) The mean difference in the total number of cells, the number of TE cells, as well as the progenitors of EPI and PE between the pairs of early twin blastocysts. (**b**) The mean difference in the percentage of TE cells, as well as the progenitors of EPI and PE between the pairs of early twin blastocysts. (**c**) The mean difference in the total number of cells, as well as the numbers of TE, PE, and EPI cells between pairs of late twin blastocysts. (**d**) The mean difference in the percentage of TE, PE, and EPI cells between the pairs of late twin blastocysts. For (**a**, **b**, **c**, and **d**), normality of the data was verified with the Kolmogorov–Smirnov test, and Student’s t-test was used for calculations. *p < 0.05, **p < 0.01, ***p < 0.001. The error bars associated with the graphs are standard deviations.

We found that in the early variation of the experiment, the pairs of twin blastocysts and pairs of control blastocysts did not differ in the total number of cells (9.8 ± 7.6 and 9.5 ± 8.5; Fig. 4a). Also, the mean differences between early twin blastocysts in the numbers of TE cells and PE progenitors were similar to those found between pairs of controls (Fig. 4a). In twins, the absolute values of the differences in the numbers of TE cells and PE progenitors were 8.6 ± 7.1 and 1.9 ± 1.3, respectively. In controls, pairs of blastocysts varied by an average of 7.8 ± 7.1 TE cells and 1.6 ± 1.4 PE progenitors. In contrast, the mean differences in the number of EPI progenitors between the twin early blastocysts were greater than between the pairs of control blastocysts (2.7 ± 2.3 and 1.9 ± 1.7; *p* = 0.04; Fig. 4a).

We also took a closer look at the cell proportions for each cell lineage (Fig. 4b). The only difference that we observed between the groups involved the EPI lineage. We noted that the variabilities between twin early blastocysts in the proportion of EPI progenitors were greater than the differences observed in the control group (7.0% ± 5.0% and 4.3% ± 3.5%; *p* = 0.0005).

Next, we compared the mean differences in the numbers of cells of the individual cell lineages between the pairs of experimental blastocysts and the pairs of control blastocysts from the late variation of the experiment (Fig. 4c). The mean differences in the total number of cells between the twin late blastocysts were more pronounced than the differences between the control blastocysts (12.1 ± 9.9 and 7.1 ± 5.3; *p* = 0.0002). Also, the mean differences in the number of TE cells between the late twin blastocysts were more substantial than between randomly matched pairs of control blastocysts (11.1 ± 8.3 and 6.9 ± 5.0; *p* = 0.0002). In turn, the mean differences in the numbers of PE and EPI cells in pairs of late experimental blastocysts and in pairs of control blastocysts were similar. In twins, pairs of blastocysts differed by an average of 2.2 ± 1.8 PE cells and 2.5 ± 2.3 EPI cells. In controls, the absolute values of the differences in the numbers of PE and EPI cells were 1.8 ± 1.4 and 2.6 ± 1.9, respectively.

We also determined whether the differences in proportions of cells of particular cell lineages between late twin blastocysts were similar to those observed between randomly selected pairs of control blastocysts. The differences between late twin blastocysts in the proportions of TE, PE, and EPI cells were greater than the differences between two control blastocysts (Fig. 4d). The pairs of experimental and control blastocysts varied by an average of 5.0% ± 4.0% and 2.5% ± 1.8% TE cells (*p* = 0.012), respectively. The mean differences in the number of PE and EPI cells between late twins were 3.0% ± 2.0% (*p* < 0.0001) and 4.0% ± 3.0% (*p* = 0.003), respectively. In controls, pairs of late blastocysts differed by an average of 1.2% ± 1.0% PE cells and 1.9% ± 1.4% EPI cells.

In summary, an analysis of twin blastocysts from the early variation of the experiment showed that these blastocysts are similar to each other. The only difference between the pairs of early twins was an imbalance in the number and proportion of EPI progenitors. In contrast, twin blastocysts from the late variation of the experiment varied considerably in both the total number of cells and the number and proportion of TE cells, as well as the percentages of EPI and PE cells. This result suggests that the differences between twin blastocysts increase over the course of development and are most pronounced at the late blastocyst stage.

### Preimplantation development of quadruplet embryos obtained by disaggregation of 4-cell embryos

#### The frequency of development to the blastocyst stage

**Figure 5:**
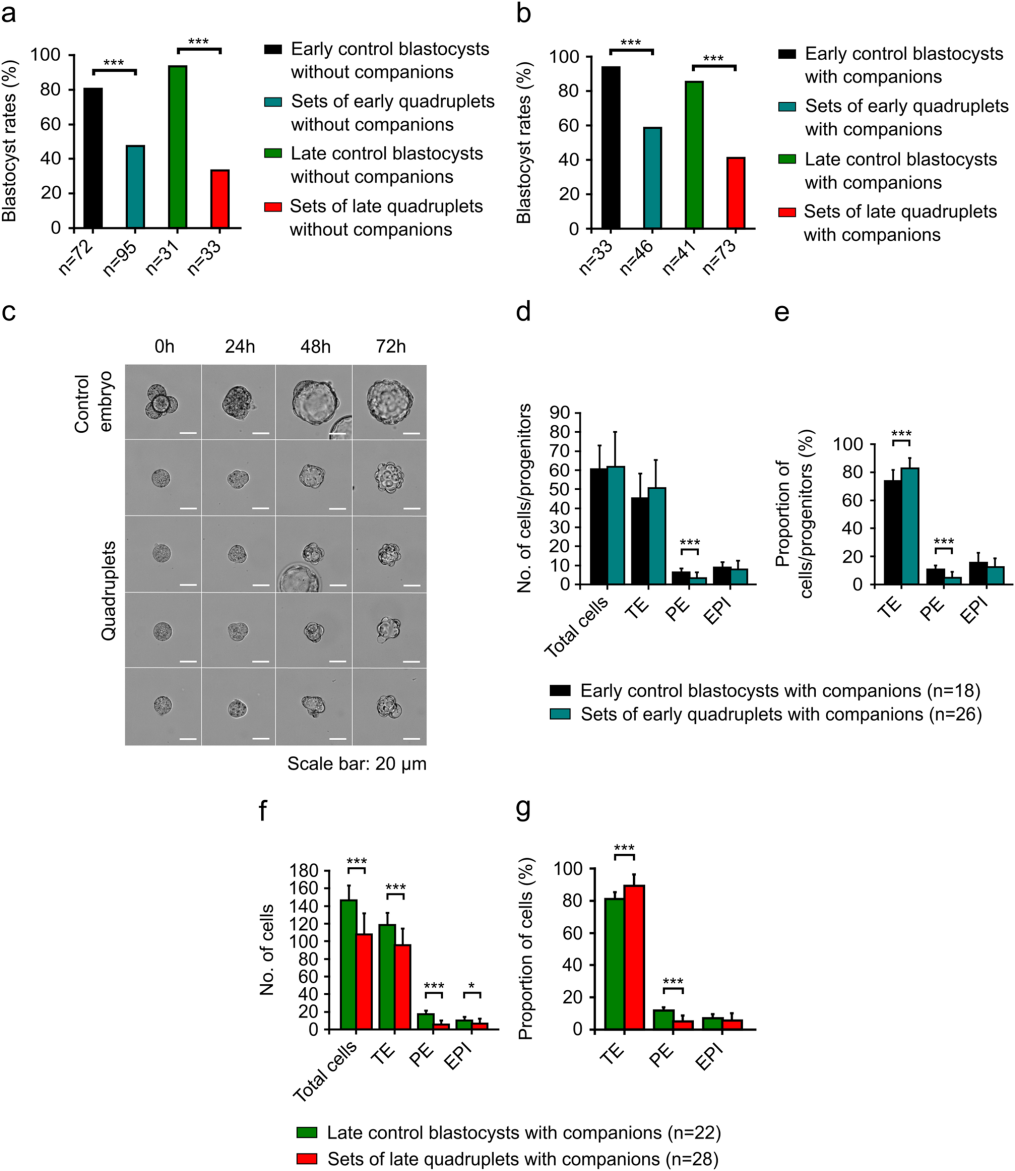
Cell lineage composition in sets of early and late quadruplet blastocysts. (**a**) Blastocyst development from 4-cell stage single blastomeres after 48 (early variation) or 72 (late variation) hours of in vitro culture without accompanying embryos. The experiments were conducted with 13 (for early variation) and 7 (for late variation) replicates. The results were compared with Chi-squared test. ***p < 0.001. (**b**) Blastocyst development from 4-cell stage single blastomeres after 48 (early variation) or 72 (late variation) hours of in vitro culture with accompanying embryos. The experiments were conducted with 6 (for early variation) and 16 (for late variation) replicates. The results were compared with Chi-squared test. ***p < 0.001. (**c**) Representative brightfield images showing embryos developed from 4-cell stage single blastomeres after 24, 48, and 72 h of in vitro culture with accompanying embryos. Scale bar: 20 µm. (**d**) Cell numbers of blastocyst lineages in sets of early quadruplet blastocysts cultured with accompanying embryos. (**e**) Percentage contribution of TE cells, as well as the progenitors of EPI and PE in sets of early quadruplet blastocysts cultured with accompanying embryos. (**f**) Average number of cells contributing to blastocyst lineages in sets of late quadruplets. (**g**) Cell numbers are shown as percentages of the total number of cells in sets of late quadruplets. For (**d**, **e**, **f**, and **g**), normality of the data was verified with the Kolmogorov–Smirnov test, and Student’s t-test was used for calculations. *p < 0.05, **p < 0.01, ***p < 0.001. Data are represented as mean ± standard deviation.

We also investigated the developmental potential of single blastomeres of a 4-cell mouse embryo to create progenitors/cells of individual cell lineages in the blastocyst. To this aim, we cultured single blastomeres and control embryos in two experimental variations. In the first variation of the experiment (“early embryos without companions”), single blastomeres of 4-cell embryos and control embryos were cultured in vitro for 48 h. As a result of disaggregation, we obtained 95 sets of quadruplets (in 13 experiments), and in 45 (47.4%) sets, all embryos in the set developed to the early blastocyst stage or formed trophoblastic vesicles (Fig. 5a). In the remaining 50 sets, we observed the lysis of at least one of the embryos, and therefore these sets were excluded from further analysis. The control group contained 72 embryos whose blastomeres were not separated at the 4-cell stage. After 48 h of culture, 58 control embryos (80.6%) reached the early blastocyst stage (Fig. 5a). The frequency of development to this stage was significantly lower in quadruplets than in the control group (47.4% *vs.* 80.6%; Chi-squared test, *p* = 0.000013).

In the second variation of the experiment (“late embryos without companions”), we cultured quadruplets and control embryos for 72 h. After disaggregation, we obtained 33 sets of quadruplets (in 7 experiments), and only in 11 (33.3%) sets did all embryos form late blastocysts or trophoblastic vesicles (Fig. 5a). In the remaining 22 sets, at least one embryo was lysed, thus these sets were excluded from further analysis. As controls, we used 31 unmanipulated 4-cell embryos, of which 29 (93.5%) progressed to the late blastocyst stage (Fig. 5a). The frequency of development to this stage was significantly lower in quadruplets than in control embryos (33.3% *vs.* 93.5%; Chi-squared test, *p* < 0.00001).

To improve the developmental rate of the quadruplets, we decided to culture them in the presence of accompanying embryos in a smaller volume of medium. Yekani et al*.* demonstrated that isolated blastomeres of 2- and 4-cell mouse embryos cultured in the presence of an intact embryo and in a small volume of medium more often reach the blastocyst stage^44,45^. Other researchers have shown that during culture, embryos communicate with each other through growth factors secreted into the environment and thus mutually stimulate their development^46–51^. Thus, we added two 4-cell accompanying embryos to each control and experimental embryo and cultured them for 48 or 72 h in 1 μl drops of medium.

In the “early embryos with companions” variation, we obtained 46 sets of quadruplets (in 6 experiments), and in 27 (58.7%) sets, all embryos formed early blastocysts or trophoblastic vesicles (Fig. 5b, c). The control group consisted of 33 embryos at the 4-cell stage, of which 31 (93.9%) reached the early blastocyst stage (Fig. 5b, c). Culture in the presence of accompanying embryos did not increase the survival of control embryos (80.6% *vs.* 93.9%) and did not improve the efficiency of obtaining complete sets of early quadruplets (47.4% *vs.* 58.7%). Despite the addition of accompanying embryos to the culture, the quadruplets had a significantly lower survival rate than the control embryos (58.7% *vs.* 93.9%; Chi-squared test, *p* = 0.0005).

In the “late embryos with companions” variation, we obtained 73 sets of quadruplets (in 16 experiments); after 72 h of culture, in 30 (41.1%) sets, all embryos formed late blastocysts or trophoblastic vesicles (Fig. 5b, c). The rate of cell lysis caused by the vigorously pipetted 4-cell stage embryos yielded 30% in both experimental variations. In the control group, 35 out of 41 (85.4%) 4-cell embryos developed to the late blastocyst stage (Fig. 5b, c). As in the “early” variation, the frequency of development of quadruplet sets was significantly lower than that of the control group (41.1% *vs.* 85.4%; Chi-squared test, *p* < 0.00001). Similarly, the presence of accompanying embryos did not increase the survival rate of the control embryos (93.5% *vs.* 85.4%) or sets of quadruplets (33.3% *vs.* 41.1%).

#### Cell lineage allocation in monozygotic quadruplet blastocysts and controls

Next, we compared sets of quadruplets that developed from single blastomeres of 4-cell embryos with control blastocysts with regard to the number and proportion of TE, EPI, and PE cells. The data for the experimental group were analyzed as the sum of the respective cells (total cells, TE, EPI or PE cells) for quadruplet blastocysts formed from a single embryo and Student’s t-test was used for all calculations. For the analysis, we used embryos from the “early embryos with companions” and “late embryos with companions” experimental variations. We analyzed:

- 18 control blastocysts that were cultured with accompanying embryos in 1 µl drops of KSOM medium (“early controls with companions” variation);
- 26 sets of quadruplets cultured with accompanying embryos in 1 µl drops of KSOM medium (“sets of early quadruplets with companions” variation);
- 22 control blastocysts that were cultured with accompanying embryos in 1 μl drops of KSOM medium (“late controls with companions” variation); and
- 28 sets of quadruplets cultured with accompanying embryos in 1 μl drops of KSOM medium (“sets of late quadruplets with companions” variation).

We found that early quadruplets and control blastocysts had a similar total number of cells (61.7 ± 18.4 *vs.* 60.4 ± 12.6; Fig. 5d; Supplementary Tables S5, S6). We also did not observe any differences in the average numbers of TE cells and EPI progenitors between the analyzed groups (Fig. 5d; Supplementary Tables S5, S6). The number of TE cells averaged 50.6 ± 14.7 in early quadruplets and 45.2 ± 12.9 in controls. In quadruplets, EPI contained on average 7.8 ± 4.6 progenitors, while in controls, their progenitors averaged 8.8 ± 3.0. However, we observed that quadruplets that developed from single blastomeres of 4-cell embryos had fewer PE progenitors than control blastocysts (3.3 ± 3.1 *vs.* 6.4 ± 2.1; *p* = 0.0006; Fig. 5d; Supplementary Tables S5, S6).

We also examined the percentages of individual cell lineages in early quadruplets and control blastocysts (Fig. 5e; Supplementary Tables S5, S6). Quadruplet blastocysts had a higher percentage of TE cells than controls (82.7% ± 7.3% *vs.* 73.7% ± 7.8%; *p* = 0.0003). In addition, the proportion of PE progenitors in the quadruplets was smaller compared to the control blastocysts (4.9% ± 4.1% *vs.* 10.7% ± 2.8%; *p* < 0.0001). In turn, the percentage of EPI progenitors was similar in both groups: 12.4% ± 6.3% in early quadruplets and 15.6% ± 7.1% in controls.

Next, we compared the composition of individual cell lineages in quadruplet and control blastocysts from the “late embryos with companions” variation (Fig. 5f; Supplementary Tables S7, S8). The total number of cells in late quadruplets was lower than in control blastocysts (107.7 ± 23.8 *vs.* 146.4 ± 16.7; *p* < 0.0001). Quadruplet embryos were also characterized by lower numbers of TE, PE, and EPI cells compared to controls. The mean numbers of TE cells of quadruplets and controls were 95.4 ± 19.2 and 118.7 ± 13.5 (*p* < 0.0001), respectively. The ICMs of late quadruplets contained on average 5.8 ± 4.3 PE cells (*p* < 0.0001) and 6.5 ± 5.7 EPI cells (*p* = 0.011), whereas the ICMs of controls averaged 17.4 ± 4.2 PE cells and 10.3 ± 4.1 EPI cells.

Then we investigated the proportions of cells in TE, PE, and EPI lineages in quadruplet and control blastocysts (Fig. 5g; Supplementary Tables S7, S8). We noted that late quadruplets had a higher fraction of TE cells (89.2% ± 7.0%) than control blastocysts (81.2% ± 4.0%; *p* < 0.0001) and that the percentage of PE cells in late quadruplet blastocysts was lower than in controls (5.1% ± 3.6% *vs.* 11.8% ± 2.0%; *p* < 0.0001). In quadruplets, the proportion of EPI cells was 5.7% ± 4.6%, which was similar to the proportion of EPI cells in control blastocysts (7.0% ± 2.6%).

Among the quadruplet blastocysts with a complete ICM, we most often observed embryos in which EPI and PE cells were segregated into appropriate positions (26 of 44 quadruplet blastocysts; 59.1%). We also noted embryos with blastomeres partly sorted in the ICMs with regard to either SOX2 or SOX17 markers (13 of 44 quadruplet blastocysts; 29.5%). We seldom observed embryos with a “salt and pepper” distribution of EPI and PE cells within the ICM (5 of 44 quadruplet blastocysts; 11.4%). In the control group, all embryos had ICM cells segregated into two distinct compartments.

To sum up, the sets of early quadruplets and controls that were cultured with accompanying embryos were similar in terms of the total number of cells, the number of TE cells, and the number and fraction of EPI progenitors. In the late variation of the experiment, the developmental potential of sets of quadruplets was notably reduced, as manifested by the negligible ability of these embryos to create particular cell lineages compared to control blastocysts.

#### Characteristics of monozygotic quadruplet blastocysts developed from one 4-cell embryo

**Figure 6:**
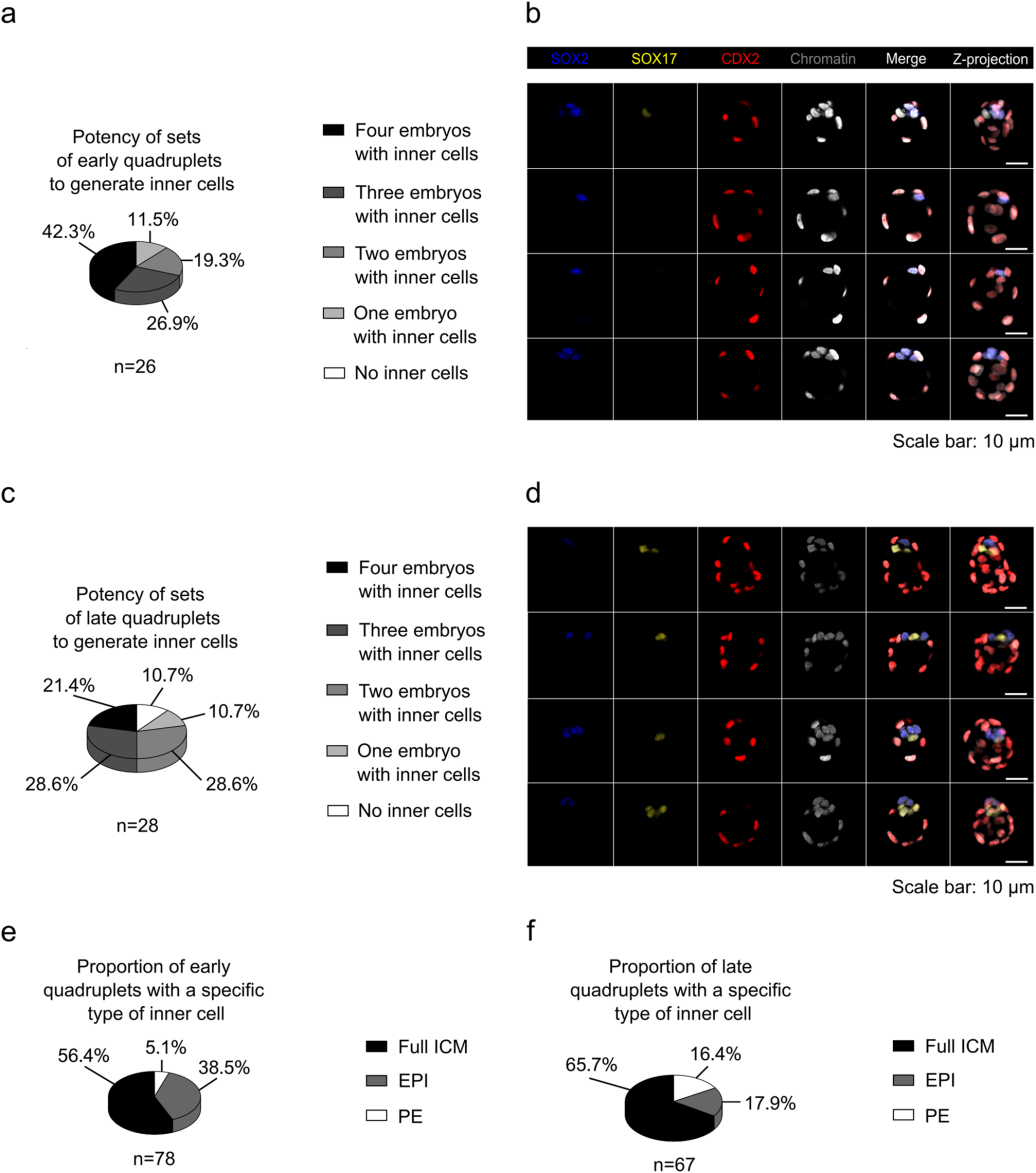
Potency of sets of early and late quadruplet blastocysts to generate inner cells. (**a**) Developmental potential of sets of early quadruplets to generate inner cells. (**b**) Representative immunofluorescence confocal images of a set of early quadruplet blastocysts showing the localization of cells expressing SOX2 (blue), SOX17 (yellow), and CDX2 (red). Chromatin (white) was labeled with chromomycin A_3_. The last panel corresponds to the average intensity projections of Z-stack images. Scale bar: 10 µm. A set in which one embryo had an ICM consisting of EPI and PE progenitors and the remaining three created only EPI progenitors is presented. (**c**) Developmental potential of sets of late quadruplets to generate inner cells. (**d**) Immunostaining for SOX2 (blue), SOX17 (yellow), and CDX2 (red) protein in a representative set of late quadruplet blastocysts. Chromatin (white) was labeled with chromomycin A_3_. The last panel corresponds to the average intensity projections of Z-stack images. Scale bar: 10 µm. A set in which four embryos had an ICM consisting of EPI and PE cells is shown. (**e**) Percentage of early quadruplets with a specific type of inner cell. *n* indicates the number of all quadruplet blastocysts (not sets). (**f**) Percentage of late quadruplets with a specific type of inner cell. *n* indicates the number of all quadruplet blastocysts (not sets).

In the next step, we compared the embryos within quadruplets derived from the blastomeres of the 4-cell embryo. We analyzed how many embryos from the set had produced at least one inner cell (EPI or PE). Among the “sets of early quadruplets with companions”, the most numerous were those in which three embryos (7 of 26 sets; 26.9%; Fig. 6a) or all (11 of 26 sets; 42.3%; Fig. 6a, b) had at least one EPI or PE progenitor. In turn, in the “sets of late quadruplets with companions” variation, we most often observed sets in which two (8 of 28 sets; 28.6%; Fig. 6c) or three (8 of 28 sets; 28.6%; Fig. 6c) embryos generated inner cells.

Then we checked the proportion of trophoblastic vesicles in each variation of the experiment. In the “early embryos with companions” variation, every fourth embryo (26 of 104 embryos; 25.0%) did not produce any inner progenitor. This was even more evident in the “late embryos with companions” variation, where 45 of 112 (40.2%) single blastomeres formed only trophoblastic vesicles.

Next, we decided to find out what kind of inner cells were present in the sets of quadruplets that developed from single blastomeres of 4-cell embryos. In the “sets of early quadruplets with companions” and “sets of late quadruplets with companions” variations, we observed 4 of 26 sets (15.4%) and 3 of 28 sets (10.7%; Fig. 6d), respectively, in which all four embryos produced ICMs consisting of both EPI and PE progenitors/cells (Supplementary Tables S9, S10). Analyzing all experimental embryos, we found that among the “early embryos with companions”, the most common were those with a complete ICM (44 of 78 embryos; 56.4%; Fig. 6e; Supplementary Table S9). Less commonly, we noted quadruplet blastocysts in which the ICM consisted only of EPI progenitors (30 of 78 embryos; 38.5%; Fig. 6e; Supplementary Table S9). Embryos had exclusively PE progenitors in the ICM much less frequently (4 of 78 embryos; 5.1%; Fig. 6e; Supplementary Table S9). In the “late embryos with companions” variation, we most often observed quadruplets that produced cells of both cell lineages in the ICM (44 of 67 embryos; 65.7%; Fig. 6f; Supplementary Table S10). The rarest were quadruplets containing only EPI cells (12 of 67 embryos; 17.9%; Fig. 6f; Supplementary Table S10) or only PE cells (11 of 67 embryos; 16.4%; Fig. 6f; Supplementary Table S10).

To investigate whether the sets of quadruplets derived from the 4-cell embryos differ in the proportion of cells in each lineage, we estimated the intraclass correlation coefficient using a random analysis of variance model^52^. For the purposes of this analysis, we introduce the term “source embryo,” which refers to the 4-cell embryo that is the source of the four sister blastomeres. We also calculated between-source variance, *i.e.,* the variability between sets of quadruplets, and within-source variance, *i.e.,* the variability between quadruplet blastocysts derived from a given set. The intraclass correlation is defined as the ratio of between-source variance to total variance and ranges from 0 to 1. If the quadruplets in the same set resemble each other in terms of the fraction of particular progenitors/cells (TE, PE, EPI), there will be no within-source variance and hence the ratio will be close to 1. Conversely, if the quadruplets from the same set are not similar, the intraclass correlation will have a value near 0.

First, we examined whether the quadruplets differ in the total number of cells and the proportion of TE cells. The intraclass correlations for the total number of cells were similar in both variations of the culture: 0.36 (*p* < 0.0001) in the “sets of early quadruplets with companions” variation and 0.27 (*p* = 0.0007) in the “sets of late quadruplets with companions” variation. This result means that the quadruplets from the same group differed in the total number of cells and that this variability was greater than the variability between sets of quadruplets. In turn, the intraclass correlation coefficient for the percentage of TE cells in the “sets of early quadruplets with companions” variation was 0.07 (*p* = 0.2012), while in the “sets of late quadruplets with companions” variation, this value was higher, at 0.25 (*p* = 0.0016). As in the case of the total number of cells, the differences in the fraction of TE cells were significantly greater between the quadruplets derived from the same 4-cell embryo than between sets of quadruplets. Then we looked at the percentages of PE and EPI progenitors/cells. The intraclass correlation coefficients for the fraction of PE progenitors/cells were 0.14 (*p* = 0.0524) in the “sets of early quadruplets with companions” variation and 0.11 (*p* = 0.0858) in the “sets of late quadruplets with companions” variation. In turn, the intraclass correlation for the percentage of EPI progenitors/cells in the “sets of early quadruplets with companions” variation was 0.11 (*p* = 0.0868), while in the “sets of late quadruplets with companions” variation it was 0.29 (*p* = 0.0004). This result suggests that the differences in the proportions of PE and EPI progenitors/cells were more pronounced between the quadruplets than between the sets of quadruplet blastocysts.

In conclusion, the vast majority of sets of quadruplets differed in their ability to generate inner cells. Some embryos within a given set had a complete ICM, while the remaining formed only EPI or PE progenitors/cells or trophoblastic vesicles. Comparing the sets of quadruplets from the “early” and “late” variations allowed us to conclude that the quadruplets from the “late embryos with companions” variation had greater potential to create an ICM composed of EPI and PE cells. These data also show that the variability between the four embryos that developed from one 4-cell embryo was greater than the variability between sets of quadruplets. The quadruplets from the “early embryos with companions” and “late embryos with companions” variations differed not only in the total number of cells but also in the proportions of PE and EPI progenitors/cells.

### Postblastocyst development of twin and quadruplet embryos derived from 2- and 4-cell stage single blastomeres

**Figure 7:**
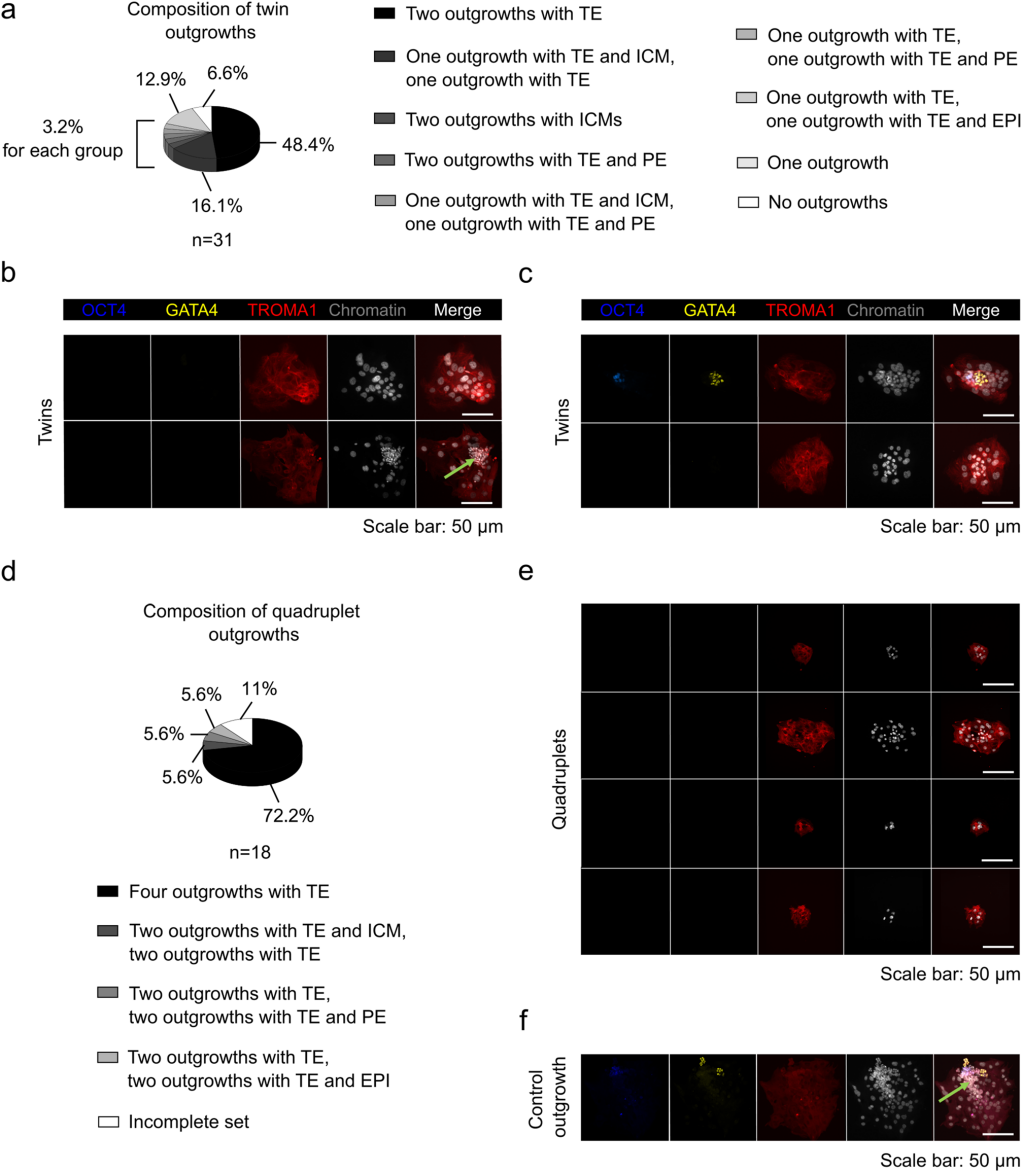
Functional analysis of twin and quadruplet blastocysts. (**a**) Composition of outgrowths derived from 2-cell stage single blastomeres. *n* indicates the number of twin pairs. (**b**) The expression of OCT4 (blue), GATA4 (yellow), and TROMA1 (red) markers in representative outgrowths developed from two sister blastomeres of a 2-cell embryo. Chromatin (white) was labeled with Hoechst 33342. Scale bar: 50 µm. A pair in which both outgrowths had only TE cells is shown. The green arrow indicates cells negative for both OCT4 and GATA4 markers. A pair in which one outgrowth created TE and ICM derivatives and the other had exclusively TE cells is presented in (**c**). (**d**) Composition of outgrowths derived from 4-cell stage single blastomeres. *n* indicates the number of quadruplet sets. (**e**) Confocal images of OCT4 (blue), GATA4 (yellow), and TROMA1 (red) expression in representative outgrowths developed from four sister blastomeres of a 4-cell embryo. Chromatin (white) was labeled with Hoechst 33342. Scale bar: 50 µm. A set in which four outgrowths composed of only TE cells is shown. A confocal image of a representative control outgrowth is presented in (**f**). The green arrow indicates cells negative for both OCT4 and GATA4 markers.

We used the blastocyst outgrowth model to evaluate the potency of the twins and quadruplets to generate all three cell lineages of the blastocyst (TE, PE, and EPI) in the outgrowths. To this aim, we transferred twin and quadruplet blastocysts into a chamber of cell culture slides coated with 0.2% gelatin and covered with embryonic stem (ES) cell medium without leukemia inhibitory factor (LIF). After 2 days in culture, the blastocysts started to form outgrowths, which we fixed on day 2 or 3, then we performed immunostaining using antibodies against OCT4 (an EPI cell marker), GATA4 (a PE cell marker), and TROMA1 (a TE cell marker). We found that 80.5% of twins (25 of 31 pairs; Fig. 7a) attached and formed two outgrowths. Among them, 48.4% of twin outgrowths (15 of 31 pairs; Fig. 7a, b) presented cells expressing only the TE cell marker. Less commonly, we observed pairs in which one outgrowth consisted of both TE and ICM derivatives and the other only TE cells (5 of 31 pairs; 16.1%; Fig. 7a, c). Most rarely, we noted twins in which both outgrowths created a complete ICM and TE cells (1 of 31 pairs; 3.2%; Fig. 7a) or pairs in which one outgrowth formed all three cell lineages and the other contained only PE and TE cells (1 of 31 pairs; 3.2%; Fig. 7a). We also observed twin outgrowths in which one outgrowth presented only TE cells and the remaining presented cells expressing TROMA1 and OCT4 (1 of 31 pairs; 3.2%; Fig. 7a) or GATA4 markers (1 of 31 pairs; 3.2%; Fig. 7a). In the remaining one case, both outgrowths had exclusively PE and TE cells (1 of 31 pairs; 3.2%; Fig. 7a).

Among the quadruplet outgrowths, 89.0% (16 of 18 sets; Fig. 7d) attached and developed four outgrowths. We most often observed sets in which the four outgrowths generated only TE cells (13 of 18 sets; 72.2%; Fig. 7d, e). In one case, we noted a set in which two outgrowths created both TE and ICM derivatives and the others only TE cells (1 of 18 sets; 5.6%; Fig. 7d). In two other cases, we observed sets in which two outgrowths presented only cells expressing TROMA1 and the others consisted of TE and EPI cells (1 of 18 sets; 5.6%; Fig. 7d) or PE cells (1 of 18 sets; 5.6%; Fig. 7d).

In addition to the OCT4- and GATA4-positive cells, we observed ICM cells that were not recognized by either antibody. These cells were noted on day 3 after blastocyst transfer to the culture dish in some twin and quadruplet outgrowths, as well as in control outgrowths (Fig. 7b, f). This phenomenon was probably due to the rapid loss of OCT4 expression that occurs in cultured rodent outgrowths prior to any evidence of morphological differentiation^53^.

Based on these data, we concluded that the functional status of twin and quadruplet outgrowths is very low. We demonstrated that blastocyst outgrowths derived from the single blastomeres of 2- and 4-cell stage embryos did not show equal competence in forming the ICM.

## Discussion

During mammalian preimplantation embryonic development, the potential of cells to differentiate into multiple lineages is gradually reduced. As development progresses, the totipotent zygote and subsequently the blastomeres arising as a result of cleavage contribute to a pluripotent EPI and two extraembryonic cell lineages: TE and PE. Despite many years of research, the exact moment when the cells of the embryo begin to differ from each other in terms of, for example, the expression of epigenetic markers or specific proteins for a particular cell lineage, is unknown. Thus, we attempted to answer the question of whether blastomeres isolated from 2- and 4-cell mouse embryo differ in their potential to create TE cells and progenitors/cells of two lineages in the ICM: EPI and PE. An advantage of our work is a new approach to the statistical characterization of the variability of twin and quadruplet blastocysts. We calculated the differences between twin blastocysts in relation to the differences between any two control embryos at the same stage of development. By using this approach, we were able to perform a more thorough analysis to determine whether the twins differ from each other in the same manner, or less or more, than randomly matched pairs of control blastocysts. We also analyzed the variability of embryos within quadruplets. Our key strategy was the ANOVA-based estimation of the intraclass correlation coefficient. This method allowed us to compare the variability within quadruplets to the variability between them and to determine which variability is more pronounced.

The blastomeres of the 2-cell mouse embryo have traditionally been considered totipotent. It has been demonstrated that single blastomeres of this stage can form a blastocyst, which after transfer to the reproductive tract of the recipient female, may develop into a normal individual^3^. This result suggested that the blastomeres of the 2-cell embryo, like the zygote, are totipotent. However, later studies that sought to obtain twin mice from one 2-cell embryo provided vague results. Monozygotic twins resulting from the development of two separated sister blastomeres are rarely born, indicating an unequal developmental potential of blastomeres of the 2-cell embryo^4–9^. This observation does not contradict the totipotency of the 2-cell mouse embryo blastomeres, but suggests uneven developmental potential of the cells emerging from the first cleavage division^8,9,54,55^.

To investigate whether sister blastomeres differ in their developmental potential, we cultured disaggregated 2-cell embryos up to the early or late blastocyst stage and analyzed the expression of markers specific for the TE, PE, and EPI lineages. We found that sister blastomeres can develop into pairs of early-stage blastocysts at a similar frequency as intact control embryos. Moreover, when we summed up the total numbers of cells in pairs of experimental monozygotic twins, they had a comparable cell number as control blastocysts at this stage. This observation is in line with those made by Katayama et al*.* and Casser et al*.* who also cultured pairs of single blastomeres of 2-cell embryos for 72 h and found that they reached the early blastocyst stage with the same frequency and were composed of a similar number of cells as control embryos^8,42^. When we compared the early twin blastocysts originating from the same embryo, they did not differ in the total number of cells or in the number and proportion of progenitors/cells of the TE and PE extraembryonic cell lineages. The only difference observed between twins at this stage related to the number and proportion of EPI progenitors, which is consistent with the report by Casser et al*.* who noted that twins differ in the ability to form EPI cells^8^. Katayama et al*.* showed that early twin blastocysts differed in both the total number of cells and the numbers of TE and ICM cells^42^. However, to identify the ICM cells, they used an antibody against the OCT4 protein, which in the early blastocyst stage is localized throughout the ICM and only later is limited to EPI cells. Therefore, it was impossible to determine whether the reduction in the number and proportion of ICM cells was due to an insufficient number of EPI progenitors. An unequal potential of the 2-cell embryo blastomeres to create EPI progenitors was also found in a study of whole intact embryos^8^. In this work, simultaneous tracing of the NANOG-expressing progeny of the sister blastomeres showed that in the vast majority of the analyzed embryos, descendants of one of the blastomeres dominated among EPI cells^8^. This result confirms that the variability in EPI composition between early twin blastocysts is not induced by the disaggregation procedure, but is due to the differences in developmental potential that already occur between blastomeres at the 2-cell stage. We found that the initial slight dissimilarity between early twin blastocysts increased as their development progressed, and at the late blastocyst stage, not only did the proportion of EPI cells differ but also the total number of cells, the number and fraction of TE cells, and the percentage of PE cells. This finding is consistent with the results of Maemura et al., who demonstrated that only 13% of 2-cell embryo blastomeres were able to form PE and EPI cell lineages in the ICM^55^. Similarly, Lorthongpanich et al*.* observed that the development of ICMs is not uniform among sister blastomeres^54^. Our results show that the blastomeres of the 2-cell embryo are potentially totipotent^3–8^; however, when compared to the zygote, their developmental potency is partly limited, which is expressed by their differing ability to produce a normal blastocyst.

The blastomeres of the mouse embryos reduce their totipotency and differ in developmental abilities beginning at the 2-cell stage. However, at this stage, at least some still retain that totipotency and after isolation can develop to term^3^. A single blastomere’s further reduction in developmental potential occurs after the next cleavage division. It has been shown that single blastomeres of a 4-cell embryo form blastocysts^10,54,55^; however, after transplantation into the oviduct of a pseudopregnant foster female, their development does not result in the birth of offspring^12^. Interestingly, a recent report demonstrated that blastomeres isolated at the 4-cell stage can develop to term, but only when the number of transferred blastocysts to pseudopregnant foster females is increased to 30 per mouse^55^. Viable pups were also obtained by Zhang et al*.,* who aggregated individual 4-cell blastomeres with supporting embryos that lack a functional TE and ICM^18^. This finding therefore indicates that a sufficient number of cells were provided for the blastomere-derived embryos to ensure their further development. Moreover, it has been demonstrated that isolated blastomeres of a 4-cell embryo form trophoblastic vesicles or small blastocysts consisting of only a few inner cells^10,54,55^. These results fall in line with our observation that the quadruplets had a limited ability to produce normal blastocysts containing all three cell lineages: TE, PE, and EPI. Only a small fraction of sets showed this potential after in vitro culture, both in the early and late experimental variations. The remaining sets of quadruplets formed trophoblastic vesicles or blastocysts containing ICMs with only one cell lineage: PE or EPI. This finding contrasts with the developmental potential of pairs of isolated 2-cell stage blastomeres, which we also analyzed in this study, and which with a high frequency formed morphologically normal early and late twin blastocysts. This result suggests that the developmental potential of these blastomeres is further reduced compared to the 2-cell stage blastomeres.

We observed that the quadruplets from the “late embryos with companions” variation had greater potential to create an ICM composed of EPI and PE cells than the quadruplets from the “early embryos with companions” variation. However, this result may be due to the companion embryos differing from each other in terms of quality. Hence, some of the blastocysts derived from the single blastomeres of a 4-cell embryo may receive better support than others. The accompanying embryos of one drop of medium may release different amounts of fibroblast growth factor 4 (FGF4) compared to other drops, which in turn would influence the EPI/PE cell balance in the blastomere-derived blastocysts. This fact might explain, at least partly, why we noted such differences between the “early” and “late” variations of the experiments.

We demonstrated that when intact control embryos reached the early blastocyst stage, blastomeres isolated at the 4-cell stage most often formed sets of quadruplets in which 3 or all embryos had at least one inner cell. The dominant types of quadruplet sets corresponding to the late control blastocysts were those in which 2 or 3 embryos generated at least one EPI or PE cell. We observed that EPI progenitors dominated in early quadruplet blastocysts, whereas in late quadruplets, EPI and PE cells occurred with a similar frequency. According to some researchers^56^, the ICM cells of late blastocysts (at the ~ 120-cell stage), in which PE and EPI cells have already been sorted and occupy their target positions (on the surface and within the ICM, respectively), maintain their plasticity and after injection into the 8-cell embryo, can differentiate into all cell lineages, including TE. Moreover, Wigger et al*.* found that in ICMs isolated from blastocysts at this stage, some cells silence and others initiate the expression of the PE-specific *Pdgfrα* gene, thus changing their developmental path from PE to EPI and *vice versa*^57^. It is possible that cells, which can still change their fate, are bipotent progenitors for both PE and EPI, coexpressing GATA6 and NANOG^58^. This suggests that similar mechanisms regulating the direction of inner cell differentiation exist in quadruplets, which may be responsible for the fact that PE progenitors emerge from cells that initially express genes characteristic of EPI. Other studies have shown that the lineage choice in the ICM may depend both on the round of asymmetric divisions in which the cell is formed and on the number of ICM progenitors generated during the first round of cell division, *i.e.,* at the transition from the 8- to 16-cell stage^59,60^. It has been suggested that the limited number of ICM progenitors formed during the first round of asymmetric divisions (at the 8- to 16-cell transition) may be insufficient to induce PE specification through the secretion of FGF4 in the remaining early ICM cells. As a result, all inner cells adopt the EPI fate and the lack of PE cells is compensated for in the next round of cell division^60^. Also, Bessonnard et al*.* reported that PE progenitors are generated later than EPI progenitors^61^. Therefore, it seems possible that a similar mechanism could work in quadruplets developing from isolated blastomeres of 4-cell embryos, thus generating mainly EPI progenitors at the early blastocyst stage and supplementing PE progenitors at the transition to the late stage.

Accordingly, we observed that quadruplets already have a notably limited ability to develop into normal blastocysts and most often form trophoblastic vesicles or blastocysts with ICMs containing only PE or EPI cells. Other researchers have also studied the developmental potential of blastomeres isolated from stages more advanced than the 2-cell stage, and by culturing single blastomeres of 4-cell embryos, they obtained trophoblastic vesicles or blastocysts comprising only a few inner cells^10,54,55^. Later, using a noninvasive cell labeling and lineage tracing method in whole 4-cell embryos, it was demonstrated that some blastomeres show developmental preferences and therefore tend to differentiate towards embryonic or extraembryonic lineages^34,40^. These results are in line with those of Guo et al*.*, who focused on the distributions of the descendants of the labeled cells at the 4-cell stage in 12.5-day-old fetuses^39^. They found that some derivatives from the labeled cells can contribute to either TE or ICM fate^39^. This result suggests that during normal development, the blastomeres of 4-cell embryos may differ in their potential to form ICM cells. The inner cells arise during three rounds of asymmetric divisions, at the 8-, 16-, and 32-cell stages^62,63^. The trophoblastic vesicles often emerge as the result of the development of isolated blastomeres of the 4-cell stage, indicating that the blastomere developing separately is subject to solely (or almost exclusively) symmetric divisions, which lead to the formation of only TE cells. This observation is consistent with the results of Humięcka et al*.*, who demonstrated that the majority of the blastomeres isolated from uncompacted 8-cell embryos preferentially undergo symmetrical divisions, resulting in the formation of embryos composed only of external cells expressing the CDX2 protein^64^. By contrast, single blastomeres of compacted 8-cell embryos often divide asymmetrically and form cells that are progenitors of both TE and ICM lineages^64^. Therefore, it seems likely that in the isolated blastomeres of 4-cell embryos, cell division regulation occurs in a similar manner as in uncompacted 8-cell embryos, thus often leading to the formation of trophoblastic vesicles.

The results of this study indicate that single blastomeres of 4-cell embryos cultured to the late blastocyst stage more often form trophoblastic vesicles than blastomeres cultured to the early blastocyst stage. The observed differences may be due to a change in the fate of inner cells, their taking up an external position, and thus contributing to the TE lineage. Grabarek et al*.* showed that such a change in fate is possible even in the case of isolated ICM cells derived from approximately 120-cell blastocysts^56^. These authors observed that after aggregation with a morulae or after injection into a morulae, ICM cells isolated from nearly 40-, 70-, and 120-cell blastocysts differentiate into TE cells, and this ability decreases with the development of the blastocyst from which they were obtained^56^.

Our observation that the twins and quadruplets have nonidentical potential to generate TE, PE, and EPI cells may be the reason for their unequal capacity to form the embryo body and fetal membranes. In our study, we also investigated the potency of sister blastomeres for multilineage differentiation in the blastocyst outgrowth assay. The outgrowth culture conditions were suboptimal due to the fact that the outgrowths were produced on gelatin coat where the extent of support is lower compared to the culture on a layer of feeder cells. We observed that nearly all of the twin and quadruplet blastocysts established two and four outgrowths, respectively, of which the majority were composed of only TE cells. Based on the expression patterns of the cell lineages markers in the blastocyst outgrowths, we revealed that twins and quadruplets have unequal potential to create PE and EPI cells. Similar observations were reported by Casser et al*.*, who transferred early twin blastocysts onto a feeder layer of mouse fetal fibroblasts to test their ability to form outgrowths^8^. They demonstrated that only slightly more than half of the twin blastocysts formed both outgrowths. Moreover, they showed that in most of the analyzed outgrowth pairs, EPI cells were absent or were present in only one outgrowth^8^. In accordance with these data, the results of Lorthongpanich et al. study also showed that sister blastomeres from 2- and 4- cell embryos differed in their developmental capability to form the ICM based on the expression pattern of the SOX2 pluripotency marker^54^. The periimplantation developmental potential of blastomere-derived blastocysts was also studied by Maemura et al.; however, in their experiments, the outgrowths were not processed together as a pair or as sets of four outgrowths and were also not subjected to immunofluorescence staining^55^. In that study, a majority of twin outgrowths manifested a normal phenotype characterized by the presence of both ICM and TE cell lineages. In contrast, the proportion of quadruplet outgrowths containing only TE cells was markedly increased^55^. These data collectively support the notion that the functional status of twin and quadruplet outgrowths is very low. Maemura et al*.* suggested that such periimplantation developmental potential might be explained by a severe defect in the ICM lineage rather than in the TE lineage^55^. Our results clearly show that the impaired formation of PE and EPI cell lineages contribute to the marked reduction in the developmental potency of blastomere-derived blastocysts. According to Morris et al*.*, the presence of at least 4 pluripotent cells within the ICM increases the chance of proper development of the embryo until birth^17^. Establishing the correct proportions of all three cell lineages in the blastocyst is therefore a prerequisite for successful embryogenesis, culminating in the birth of an animal.

Taken together, our results demonstrate that the developmental potential of 4-cell stage blastomeres is more limited than that of 2-cell stage blastomeres. The quadruplets, unlike twins, quite often fail to develop into normal blastocysts and form trophoblastic vesicles or blastocysts with ICMs containing cells of only one cell lineage: PE or EPI. Our study also shows that both the 2- and 4-cell stage blastomeres differ in their individual developmental abilities to create embryonic and extraembryonic cell lineages. These findings may have relevance for the application of the embryo splitting/disaggregation method to increase the number of human embryos available for intrauterine transfer in reproductive medicine.

## Methods

### Animals

Wild-type F1 (C57BL/6/Tar x CBA/Tar) mice were used in this study. All animal experiments were approved by the Local Ethics Committee for Experimentation on Animals no. 1 (Warsaw, Poland) and were conducted in accordance with the ARRIVE guidelines and national regulations.

### Experimental procedures

#### Embryo collection

Embryos for blastomere isolation at the 2- and 4-cell stages were collected from naturally mated F1 mice. Embryos at noon on the day of detecting copulatory plugs were considered to be E0.5. Experimental embryos corresponding to E1.5 (the 2-cell stage) and E2.0 (the 4-cell stage) were recovered from isolated oviducts and placed into M2 medium supplemented with 4 mg/ml bovine serum albumin^65^ (BSA, Sigma-Aldrich). Supporting 4-cell stage embryos were obtained from F1 females superovulated by an intraperitoneal injection of 10 IU pregnant mare serum gonadotropin (PMSG, Intervet), followed by 10 IU human chorionic gonadotropin (hCG, Intervet) 44–52 h later. The female mice with vaginal plugs were autopsied, and their dissected oviducts were flushed using M2 medium with BSA at 54 h after hCG injection.

#### Embryo disaggregation and in vitro culture of blastomeres

The *zona pelucida* was removed using acidic Tyrode’s solution^66^ (10–30 s of treatment depending on the temperature of the solution). The embryos were then placed in M2 medium with BSA and incubated at 37.5 °C in 5% CO_2_ in the air. After 15 min of culture in M2 medium, the embryos were transferred to Ca^2+^ and Mg^2+^-free M2 medium supplemented with ethylene glycol-bis(β-aminoethyl ether)-N,N,N′,N′-tetraacetic acid (EGTA, 0.2 mg/ml, Sigma-Aldrich) and incubated for 15 min before disaggregation. Embryos at the 2-cell stage were subsequently dissociated into single cells with a thin glass needle. Embryos at the 4-cell stage were disaggregated by vigorous pipetting. Control embryos were subjected to the same experimental steps as the twins and quadruplets, but their blastomeres were not separated. The pairs of single blastomeres and the control embryos were cultured individually in 5 µl of KSOM medium^67^ (Merck Millipore) in standard conditions (37.5 °C, 5% CO_2_ in the air). The sets of four single blastomeres were cultured in two variations. Initially, quadruplets were cultured individually in 5 µl droplets. To improve blastocyst development from single blastomeres, we added two supporting 4-cell embryos to each individual blastomere and control embryo and decreased the medium volume from 5 to 1 µl. Some sets of two and four blastomeres were cultured for 72 and 48 h, respectively, until they reached the stage corresponding to the early blastocyst (typified by “salt and pepper” distribution of EPI and PE progenitors within the ICM^68–71^) in control groups. Other sets of two and four blastomeres derived from single embryos were cultured for 96 and 72 h, respectively. At that time, control embryos developed to the late blastocyst stage were composed of EPI and PE cells segregated in ICMs.

#### Blastocyst outgrowth formation

For blastocyst outgrowth formation assays, late twin and quadruplet blastocysts (96 and 72 h of culture, respectively) were cultured in pairs or sets of four in ES medium without LIF in 8 well cell culture slides (MatTek Corporation) pre-coated with 0.2% gelatin (Sigma-Aldrich). The ES medium contained knockout DMEM (Dulbecco Modified Eagle Medium, Thermo Fisher Scientific) supplemented with 15% FBS (fetal bovine serum, Thermo Fisher Scientific), streptomycin (50 µg /ml, Thermo Fisher Scientific), penicillin (50 units/ml, Thermo Fisher Scientific), nonessential aminoacids (0.1 mM, Thermo Fisher Scientific), l-glutamine (2 mM, Thermo Fisher Scientific), and β-mercaptoethanol (0.1 mM, Sigma-Aldrich). Outgrowths were cultured for 48–72 h and subsequently fixed and stained for OCT4, GATA4, and TROMA1.

#### Immunostaining

Although sister embryos were cultured individually, they were always processed together as a pair or sets of four blastocysts. Twin blastocysts, quadruplet blastocysts, and their cultured outgrowths were fixed in 4% paraformaldehyde (Thermo Fisher Scientific) in Ca^2+^ and Mg^2+^-free PBS (phosphate-buffered saline, Biomed) for 30 min at room temperature, treated with 0.5% Triton X-100 (Sigma-Aldrich) in Ca^2+^ and Mg^2+^-free PBS for 30 min at room temperature, and placed in PBS containing 10% FBS (Thermo Fisher Scientific) or 3% BSA (Sigma-Aldrich), and 0.01% sodium azide (Honeywell Fluka) overnight at 4 °C. The following primary antibodies were used to analyze cell lineage allocation: mouse monoclonal antibody against CDX2 (1:50, BioGenex, MU392A-UC), rabbit polyclonal antibody against SOX2 (1:100, Abcam, ab97959), goat polyclonal antibody against SOX17 (1:100, R&D Systems, AF1924), mouse monoclonal antibody against OCT4 (1:100, Santa Cruz Biotechnology, sc-5279), goat polyclonal antibody against GATA4 (1:100, R&D Systems, AF2606), and rat monoclonal antibody against TROMA1 (1:50, Developmental Studies Hybridoma Bank, AB_531826). After 24 h of incubation at 4 °C, the blastocysts and their outgrowths were washed three times for 15 min each in Ca^2+^ and Mg^2+^-free PBS and then incubated with secondary antibodies: Alexa Fluor 594-conjugated donkey anti-mouse IgG (1:200, Invitrogen, A21203), Alexa Fluor 647-conjugated donkey anti-rabbit (1:200, Invitrogen, A31573), Alexa Fluor 488-conjugated donkey anti-goat IgG (1:200, Invitrogen, A11055), and Alexa Fluor 633-conjugated goat anti-rat IgG (1:200, Invitrogen, A21094) for 1 h at room temperature and washed again in PBS. Antibodies were diluted in blocking solution. To visualize the nuclei, the blastocysts were stained in microdroplets of chromomycin A_3_ (0.01 mg/ml with 5 mM MgCl_2_ in PBS, Sigma-Aldrich) on glass-bottom dishes (MatTek Corporation) for 30 min at 37.5 °C. The outgrowths were stained with Hoechst 33342 (20 µg/ml in PBS, Riedel-de Haën) for 20 min at 37.5 °C, then washed three times for 15 min each in PBS. After removing the final PBS, the outgrowths were kept slightly wet and then coverslipped with a small volume of CitiFluor AF1 (Glycerol/PBS solution, Science Services). Images of the embryos were taken on a Zeiss LSM 510 laser scanning confocal microscope and analyzed with ImageJ software (version 1.52a). The fluorescence images of the blastocyst outgrowths were acquired with a Nikon Ti2-U microscope with focus motor assembly (Prior Scientific Instruments) using the rescan confocal microscopy module RCM1 (Confocal.nl) with an ORCA-Flash4.0 LT + camera (Hamamatsu) and iChrome CLE 50 laser engine (Toptica), all controlled by μManager software^72,73^. The confocal fluorescence images were captured with a CFI Plan Apochromat Lambda S 10X objective (Nikon) and then analyzed using the ZEN 2.3 (blue edition) and ImageJ software programs.

### Statistical analysis

We conducted statistical analyses using IBM SPSS Statistics 23. We analyzed the basic descriptive statistics together with the Kolomogorov-Smirnov test, the Chi-squared test, and the Student’s t-test for independent samples. We calculated the total number of cells, as well as the number and fractions of TE, EPI, and PE cells in the embryos from the control and experimental groups. In the calculations performed for the experimental embryos, we took into account the sum of the number of cells for a given pair of twin embryos or a set of quadruplet blastocysts. *P* values of less than 0.05 were considered statistically significant. The number that follows the ± sign is a standard deviation.

#### Analysis of the differences between twin blastocysts in relation to the differences between any two control embryos at the same stage of development

To determine whether the differences in the number of cells of a particular cell line between twin blastocysts are comparable to the normally observed differences between two control embryos at the same stage of development, we calculated the absolute value of half the difference in the number of cells of each cell line for 1465 and 1400 randomly selected pairs of early and late control embryos, respectively. From this calculation, we obtained the reference values of the mean and standard deviation for the embryos cultured under control conditions. We then compared the results obtained for twin blastocysts to the reference values to see if they were identical to the results of the control group. For this purpose, we used the Student’s t-test for one mean. *P* < 0.05 was considered statistically significant.

#### Analysis of the variability of embryos within quadruplets

To quantify the similarity of source embryos with respect to the fraction of particular cells (TE, PE, or EPI) present in sister embryos, we used the intraclass correlation coefficient^74,75^. This coefficient is the ratio of estimates of between-source variance to total variance; within-source variance is estimated by MS_*within*_ from ANOVA, between-source variance by (MS_*between*_ − MS_*within*_)/4, and total variance by the sum of the two. MS is an abbreviation for mean square. The intraclass correlation takes values ranging from 0 to 1; the higher the similarity of the sister embryos within their source, the higher the value. However, the similarity of sources exceeding that which would be expected under a random model, resulting in an excess of relative dissimilarity of embryos within quadruplets, would yield negative values. ANOVA-based estimates of intraclass correlation and their statistical significance^52^ are presented.

## Supplementary Information

Supplementary Tables.

## References

[1] Condic ML (2014). Totipotency: What it is and what it is not. Stem Cells Dev..

[2] Klimczewska K, Kasperczuk A, Suwińska A (2018). The regulative nature of mammalian embryos. Curr. Top. Dev. Biol..

[3] Tarkowski AK (1959). Experiments on the development of isolated blastomers of mouse eggs. Nature.

[4] Mullen, R., Whitten, W. & Carter, S. Studies on chimeric mice and half-embryos. In *Annual Report of the Jackson Laboratory* 67–68 (Jackson Laboratory, 1970).

[5] Tsunoda Y, McLaren A (1983). Effect of various procedures on the viability of mouse embryos containing half the normal number of blastomeres. J. Reprod. Fertil..

[6] Papaioannou VE, Mkandawire J, Biggers JD (1989). Development and phenotypic variability of genetically identical half mouse embryos. Dev. Camb. Engl..

[7] Papaioannou VE, Ebert KM (1995). Mouse half embryos: Viability and allocation of cells in the blastocyst. Dev. Dyn. Off. Publ. Am. Assoc. Anat..

[8] Casser E (2017). Totipotency segregates between the sister blastomeres of two-cell stage mouse embryos. Sci. Rep..

[9] Boiani M, Casser E, Fuellen G, Christians ES (2019). Totipotency continuity from zygote to early blastomeres: A model under revision. Reprod. Camb. Engl..

[10] Tarkowski AK, Wróblewska J (1967). Development of blastomeres of mouse eggs isolated at the 4- and 8-cell stage. J. Embryol. Exp. Morphol..

[11] Kelly, S. J. Studies of the potency of the early cleavage blastomeres of the mouse. In *The Early Development of Mammals* 97–105 (Cambridge University Press, 1975).

[12] Rossant J (1976). Postimplantation development of blastomeres isolated from 4- and 8-cell mouse eggs. J. Embryol. Exp. Morphol..

[13] Kelly SJ (1977). Studies of the developmental potential of 4- and 8-cell stage mouse blastomeres. J. Exp. Zool..

[14] Tarkowski AK, Ozdzenski W, Czołowska R (2001). Mouse singletons and twins developed from isolated diploid blastomeres supported with tetraploid blastomeres. Int. J. Dev. Biol..

[15] Tarkowski AK, Ozdzenski W, Czolowska R (2005). Identical triplets and twins developed from isolated blastomeres of 8- and 16-cell mouse embryos supported with tetraploid blastomeres. Int. J. Dev. Biol..

[16] Tarkowski AK, Suwińska A, Czołowska R, Ożdżeński W (2010). Individual blastomeres of 16- and 32-cell mouse embryos are able to develop into foetuses and mice. Dev. Biol..

[17] Morris SA, Guo Y, Zernicka-Goetz M (2012). Developmental plasticity is bound by pluripotency and the Fgf and Wnt signaling pathways. Cell Rep..

[18] Zhang X (2018). Individual blastomeres of 4- and 8-cell embryos have ability to develop into a full organism in mouse. J. Genet. Genom. Yi Chuan Xue Bao.

[19] Roberts RM, Katayama M, Magnuson SR, Falduto MT, Torres KEO (2011). Transcript profiling of individual twin blastomeres derived by splitting two-cell stage murine embryos. Biol. Reprod..

[20] Biase FH, Cao X, Zhong S (2014). Cell fate inclination within 2-cell and 4-cell mouse embryos revealed by single-cell RNA sequencing. Genome Res..

[21] Shi J (2015). Dynamic transcriptional symmetry-breaking in pre-implantation mammalian embryo development revealed by single-cell RNA-seq. Dev. Camb. Engl..

[22] Casser E, Israel S, Schlatt S, Nordhoff V, Boiani M (2018). Retrospective analysis: Reproducibility of interblastomere differences of mRNA expression in 2-cell stage mouse embryos is remarkably poor due to combinatorial mechanisms of blastomere diversification. Mol. Hum. Reprod..

[23] Hupalowska A (2018). CARM1 and paraspeckles regulate pre-implantation mouse embryo development. Cell.

[24] Wang J (2018). Asymmetric expression of LincGET biases cell fate in two-cell mouse embryos. Cell.

[25] Torres-Padilla M-E, Parfitt D-E, Kouzarides T, Zernicka-Goetz M (2007). Histone arginine methylation regulates pluripotency in the early mouse embryo. Nature.

[26] Burton A (2013). Single-cell profiling of epigenetic modifiers identifies PRDM14 as an inducer of cell fate in the mammalian embryo. Cell Rep..

[27] Goolam M (2016). Heterogeneity in Oct4 and Sox2 targets biases cell fate in 4-cell mouse embryos. Cell.

[28] Jedrusik A (2008). Role of Cdx2 and cell polarity in cell allocation and specification of trophectoderm and inner cell mass in the mouse embryo. Genes Dev..

[29] Plachta N, Bollenbach T, Pease S, Fraser SE, Pantazis P (2011). Oct4 kinetics predict cell lineage patterning in the early mammalian embryo. Nat. Cell Biol..

[30] White MD (2016). Long-lived binding of Sox2 to DNA predicts cell fate in the four-cell mouse embryo. Cell.

[31] Piotrowska K, Wianny F, Pedersen RA, Zernicka-Goetz M (2001). Blastomeres arising from the first cleavage division have distinguishable fates in normal mouse development. Dev. Camb. Engl..

[32] Gardner RL (2007). The axis of polarity of the mouse blastocyst is specified before blastulation and independently of the zona pellucida. Hum. Reprod. Oxf. Engl..

[33] Alarcón VB, Marikawa Y (2003). Deviation of the blastocyst axis from the first cleavage plane does not affect the quality of mouse postimplantation development. Biol. Reprod..

[34] Fujimori T, Kurotaki Y, Miyazaki J-I, Nabeshima Y-I (2003). Analysis of cell lineage in two- and four-cell mouse embryos. Dev. Camb. Engl..

[35] Chróścicka A, Komorowski S, Maleszewski M (2004). Both blastomeres of the mouse 2-cell embryo contribute to the embryonic portion of the blastocyst. Mol. Reprod. Dev..

[36] Alarcón VB, Marikawa Y (2005). Unbiased contribution of the first two blastomeres to mouse blastocyst development. Mol. Reprod. Dev..

[37] Motosugi N, Bauer T, Polanski Z, Solter D, Hiiragi T (2005). Polarity of the mouse embryo is established at blastocyst and is not prepatterned. Genes Dev..

[38] Waksmundzka M, Wisniewska A, Maleszewski M (2006). Allocation of cells in mouse blastocyst is not determined by the order of cleavage of the first two blastomeres. Biol. Reprod..

[39] Guo S (2020). Tracing the origin of the placental trophoblast cells in mouse embryo development†. Biol. Reprod..

[40] Tabansky I (2013). Developmental bias in cleavage-stage mouse blastomeres. Curr. Biol. CB.

[41] Piotrowska-Nitsche K, Perea-Gomez A, Haraguchi S, Zernicka-Goetz M (2005). Four-cell stage mouse blastomeres have different developmental properties. Dev. Camb. Engl..

[42] Katayama M, Ellersieck MR, Roberts RM (2010). Development of monozygotic twin mouse embryos from the time of blastomere separation at the two-cell stage to blastocyst. Biol. Reprod..

[43] Chazaud C, Yamanaka Y (2016). Lineage specification in the mouse preimplantation embryo. Dev. Camb. Engl..

[44] Yekani F, Azarnia M, Esfandiari F, Hassani S-N, Baharvand H (2018). Enhanced development of mouse single blastomeres into blastocysts via the simultaneous inhibition of TGF-β and ERK pathways in microdroplet culture. J. Cell. Biochem..

[45] Yekani F (2018). Enhancing developmental rate and quality of mouse single blastomeres into blastocysts using a microplatform. J. Cell. Physiol..

[46] Paria BC, Dey SK (1990). Preimplantation embryo development in vitro: Cooperative interactions among embryos and role of growth factors. Proc. Natl. Acad. Sci. U. S. A..

[47] Brison DR, Schultz RM (1997). Apoptosis during mouse blastocyst formation: Evidence for a role for survival factors including transforming growth factor alpha. Biol. Reprod..

[48] Kelley RL, Gardner DK (2016). Combined effects of individual culture and atmospheric oxygen on preimplantation mouse embryos in vitro. Reprod. Biomed. Online.

[49] Kelley RL, Gardner DK (2017). Addition of interleukin-6 to mouse embryo culture increases blastocyst cell number and influences the inner cell mass to trophectoderm ratio. Clin. Exp. Reprod. Med..

[50] Hardy K, Spanos S (2002). Growth factor expression and function in the human and mouse preimplantation embryo. J. Endocrinol..

[51] Wydooghe E (2017). Autocrine embryotropins revisited: How do embryos communicate with each other in vitro when cultured in groups?. Biol. Rev. Camb. Philos. Soc..

[52] Neter J, Wasserman W, Kutner M (1985). Applied Linear Statistical Models.

[53] Buehr M (2003). Rapid loss of Oct-4 and pluripotency in cultured rodent blastocysts and derivative cell lines. Biol. Reprod..

[54] Lorthongpanich C, Yang S-H, Piotrowska-Nitsche K, Parnpai R, Chan AWS (2008). Development of single mouse blastomeres into blastocysts, outgrowths and the establishment of embryonic stem cells. Reprod. Camb. Engl..

[55] Maemura M (2021). Totipotency of mouse zygotes extends to single blastomeres of embryos at the four-cell stage. Sci. Rep..

[56] Grabarek JB (2012). Differential plasticity of epiblast and primitive endoderm precursors within the ICM of the early mouse embryo. Dev. Camb. Engl..

[57] Wigger M (2017). Plasticity of the inner cell mass in mouse blastocyst is restricted by the activity of FGF/MAPK pathway. Sci. Rep..

[58] Saiz N, Williams KM, Seshan VE, Hadjantonakis A-K (2016). Asynchronous fate decisions by single cells collectively ensure consistent lineage composition in the mouse blastocyst. Nat. Commun..

[59] Morris SA, Graham SJL, Jedrusik A, Zernicka-Goetz M (2013). The differential response to Fgf signalling in cells internalized at different times influences lineage segregation in preimplantation mouse embryos. Open Biol..

[60] Krupa M (2014). Allocation of inner cells to epiblast vs primitive endoderm in the mouse embryo is biased but not determined by the round of asymmetric divisions (8→16- and 16→32-cells). Dev. Biol..

[61] Bessonnard S (2017). ICM conversion to epiblast by FGF/ERK inhibition is limited in time and requires transcription and protein degradation. Sci. Rep..

[62] Morris SA (2010). Origin and formation of the first two distinct cell types of the inner cell mass in the mouse embryo. Proc. Natl. Acad. Sci. U. S. A..

[63] Humięcka M, Krupa M, Guzewska MM, Maleszewski M, Suwińska A (2016). ESCs injected into the 8-cell stage mouse embryo modify pattern of cleavage and cell lineage specification. Mech. Dev..

[64] Humięcka M, Szpila M, Kłoś P, Maleszewski M, Szczepańska K (2017). Mouse blastomeres acquire ability to divide asymmetrically before compaction. PLoS ONE.

[65] Fulton BP, Whittingham DG (1978). Activation of mammalian oocytes by intracellular injection of calcium. Nature.

[66] Nicolson GL, Yanagimachi R, Yanagimachi H (1975). Ultrastructural localization of lectin-binding sites on the zonae pellucidae and plasma membranes of mammalian eggs. J. Cell Biol..

[67] Erbach GT, Lawitts JA, Papaioannou VE, Biggers JD (1994). Differential growth of the mouse preimplantation embryo in chemically defined media. Biol. Reprod..

[68] Chazaud C, Yamanaka Y, Pawson T, Rossant J (2006). Early lineage segregation between epiblast and primitive endoderm in mouse blastocysts through the Grb2-MAPK pathway. Dev. Cell.

[69] Plusa B, Piliszek A, Frankenberg S, Artus J, Hadjantonakis A-K (2008). Distinct sequential cell behaviours direct primitive endoderm formation in the mouse blastocyst. Dev. Camb. Engl..

[70] Guo G (2010). Resolution of cell fate decisions revealed by single-cell gene expression analysis from zygote to blastocyst. Dev. Cell.

[71] Bessonnard S (2014). Gata6, Nanog and Erk signaling control cell fate in the inner cell mass through a tristable regulatory network. Dev. Camb. Engl..

[72] Edelstein A, Amodaj N, Hoover K, Vale R, Stuurman N (2010). Computer control of microscopes using µManager. Curr. Protoc. Mol. Biol..

[73] Edelstein AD (2014). Advanced methods of microscope control using μManager software. J. Biol. Methods.

[74] Cox DR, Solomon PJ (2003). Components of Variance.

[75] Rao PSRS (1997). Variance Components Estimation.

