# Supplementary material for: Developmental capacity is unevenly distributed among single blastomeres of 2-cell and 4-cell stage mouse embryos
Source: Sci Rep. 2021 Nov 2;11:21422. doi: 10.1038/s41598-021-00834-1 (PMC8563712; doi:10.1038/s41598-021-00834-1)
Supplement: Supplementary file 1 — Supplementary Tables. [file 41598_2021_834_MOESM1_ESM.pdf]

Supplementary Information

Developmental capacity is unevenly distributed among single blastomeres of 2-cell and 4-cell stage mouse embryos

Katarzyna Krawczyk[1*], Ewa Kosyl[1], Karolina Częścik-Łysyszyn[1], Tomasz Wyszomirski[2] and Marek Maleszewski[1*]

[1]Department of Embryology, Institute of Developmental Biology and Biomedical Sciences, Faculty of Biology, University of Warsaw, Miecznikowa 1, 02-096 Warsaw, Poland

[2]Department of Ecology and Environmental Protection, Institute of Environmental Biology, Faculty of Biology, University of Warsaw, Żwirki i Wigury 101, 02-089 Warsaw, Poland

[*]Corresponding authors: Katarzyna Krawczyk (k.klimczewska@biol.uw.edu.pl), Marek Maleszewski (maleszewski@biol.uw.edu.pl)

Supplementary Table S1. Descriptive statistics of the quantitative variables of early control blastocysts.

n=54	*Mean*	*Median*	*Standard deviation*	*Skewness*	*Minimum*	*Maximum*	*p*
Total cells	78.25	77.00	14.48	0.47	54.00	123.00	0.147
TE	61.56	60.50	12.49	0.60	38.00	105.00	>0.15
PE	8.65	8.00	2.76	0.76	3.00	16.00	<0.01
EPI	8.04	7.00	3.54	1.65	3.00	23.00	<0.01
Proportion of TE	0.79	0.79	0.05	-0.76	0.64	0.86	>0.15
Proportion of PE	0.11	0.11	0.03	0.42	0.04	0.20	>0.15
Proportion of EPI	0.10	0.10	0.04	0.91	0.04	0.22	>0.15

Data correspond to those in Figure 1c and 1d. The *p* values were determined by Kolmogorov-Smirnov test.

Supplementary Table S2. Descriptive statistics of the quantitative variables of pairs of early twin blastocysts.

n=40	*Mean*	*Median*	*Standard deviation*	*Skewness*	*Minimum*	*Maximum*	*p*
Total cells	78.29	78.50	21.97	-0.02	31.00	129.00	>0.15
TE	64.28	62.50	18.89	0.01	24.00	108.00	>0.15
PE	6.18	5.00	3.19	0.68	1.00	14.00	<0.01
EPI	7.83	8.00	3.18	0.06	1.00	16.00	>0.15
Proportion of TE	0.82	0.82	0.05	0.07	0.72	0.92	>0.15
Proportion of PE	0.08	0.08	0.04	0.76	0.02	0.18	>0.15
Proportion of EPI	0.10	0.10	0.04	0.13	0.02	0.19	>0.15

Data correspond to those in Figure 1c and 1d. The *p* values were determined by Kolmogorov-Smirnov test.

Supplementary Table S3. Descriptive statistics of the quantitative variables of late control blastocysts.

n=30	*Mean*	*Median*	*Standard deviation*	*Skewness*	*Minimum*	*Maximum*	*p*
Total cells	136. 37	134.50	12.92	0.08	114.00	160.00	>0.15
TE	110.73	112.00	12.45	-0.07	88.00	133.00	>0.15
PE	14.07	13.50	3.20	0.35	9.00	21.00	>0.15
EPI	11.57	11.00	4.69	0.48	4.00	21.00	0.126
Proportion of TE	0.81	0.81	0.04	0.09	0.73	0.90	>0.15
Proportion of PE	0.10	0.10	0.02	0.60	0.06	0.16	>0.15
Proportion of EPI	0.09	0.08	0.03	0.42	0.03	0.16	>0.15

Data correspond to those in Figure 1e and 1f. The *p* values were determined by Kolmogorov-Smirnov test.

Supplementary Table S4. Descriptive statistics of the quantitative variables of pairs of late twin blastocysts.

n=61	*Mean*	*Median*	*Standard deviation*	*Skewness*	*Minimum*	*Maximum*	*p*
Total cells	117.90	120.00	23.58	-1.19	42.00	156.00	0.079
TE	99.92	103.00	19.80	-0.75	42.00	134.00	>0.15
PE	9.08	9.00	4.32	0.12	0.00	19.00	>0.15
EPI	8.90	9.00	5.49	0.51	0.00	26.00	0.085
Proportion of TE	0.85	0.85	0.07	-0.17	0.66	1.00	>0.15
Proportion of PE	0.08	0.07	0.03	-0.11	0.00	0.14	>0.15
Proportion of EPI	0.07	0.08	0.04	0.45	0.00	0.20	>0.15

Data correspond to those in Figure 1e and 1f. The *p* values were determined by Kolmogorov-Smirnov test.

Supplementary Table S5. Descriptive statistics of the quantitative variables of early control blastocysts cultured with accompanying embryos.

n=18	*Mean*	*Median*	*Standard deviation*	*Skewness*	*Minimum*	*Maximum*	*p*
Total cells	60.44	61.00	12.63	-0.06	41.00	81.00	>0.15
TE	45.22	47.50	12.88	-0.08	25.00	65.00	>0.15
PE	6.39	6.0	2.06	0.18	3.00	10.00	0.097
EPI	8.83	9.50	3.00	-0.04	4.00	14.00	>0.15
Proportion of TE	0.74	0.75	0.08	-0.47	0.59	0.84	>0.15
Proportion of PE	0.11	0.11	0.03	-0.58	0.05	0.15	>0.15
Proportion of EPI	0.15	0.16	0.07	0.33	0.05	0.30	>0.15

Data correspond to those in Figure 5d and 5e. The *p* values were determined by Kolmogorov-Smirnov test.

Supplementary Table S6. Descriptive statistics of the quantitative variables of sets of early quadruplets cultured with accompanying embryos.

n=26	*Mean*	*Median*	*Standard deviation*	*Skewness*	*Minimum*	*Maximum*	*p*
Total cells	61.65	62.50	18.40	0.18	27.00	104.00	>0.15
TE	50.60	46.50	14.75	0.40	23.00	87.00	>0.15
PE	3.27	3.00	3.08	1.25	0.00	12.00	0.037
EPI	7.78	7.50	4.62	-0.01	1.00	15.00	0.06
Proportion of TE	0.83	0.82	0.07	0.34	0.72	0.97	>0.15
Proportion of PE	0.05	0.04	0.04	0.89	0.00	0.15	0.136
Proportion of EPI	0.12	0.13	0.06	-0.21	0.02	0.22	>0.15

Data correspond to those in Figure 5d and 5e. The *p* values were determined by Kolmogorov-Smirnov test.

Supplementary Table S7. Descriptive statistics of the quantitative variables of late control blastocysts cultured with accompanying embryos.

n=22	*Mean*	*Median*	*Standard deviation*	*Skewness*	*Minimum*	*Maximum*	*p*
Total cells	146.36	141.50	16.69	0.15	118.00	173.00	>0.15
TE	118.68	118.00	13.52	0.28	97.00	144.00	>0.15
PE	17.39	16.50	4.19	0.67	10.00	27.00	>0.15
EPI	10.29	9.50	4.12	0.64	4.00	19.00	0.13
Proportion of TE	0.81	0.81	0.04	-0.10	0.73	0.89	>0.15
Proportion of PE	0.12	0.11	0.02	0.24	0.07	0.16	>0.15
Proportion of EPI	0.07	0.06	0.03	0.32	0.03	0.12	>0.15

Data correspond to those in Figure 5f and 5g. The *p* values were determined by Kolmogorov-Smirnov test.

Supplementary Table S8. Descriptive statistics of the quantitative variables of sets of late quadruplets cultured with accompanying embryos.

n=28	*Mean*	*Median*	*Standard deviation*	*Skewness*	*Minimum*	*Maximum*	*p*
Total cells	107.68	104.50	23.83	0.49	70.00	164.00	>0.15
TE	95.43	93.00	19.19	0.39	66.00	139.00	>0.15
PE	5.79	6.00	4.31	0.33	0.00	16.00	>0.15
EPI	6.46	6.00	5.74	0.81	0.00	22.00	>0.15
Proportion of TE	0.89	0.89	0.07	-0.20	0.75	1.00	>0.15
Proportion of PE	0.05	0.06	0.04	0.09	0.00	0.13	>0.15
Proportion of EPI	0.06	0.06	0.05	0.36	0.00	0.16	>0.15

Data correspond to those in Figure 5f and 5g. The *p* values were determined by Kolmogorov-Smirnov test.

Supplementary Table S9. Developmental potential of sets of early quadruplets to generate inner cells. Abbreviations: EPI – an embryo that produced only EPI progenitors; PE – an embryo that generated only PE progenitors; ICM – an embryo that developed an ICM composed of two lineages: EPI and PE. The number of sets of early quadruplets with a given type of inner cell is given in parentheses.

**No inner cells**	**One embryo with inner cells**	**Two embryos with inner cells**	**Three embryos with inner cells**	**Four embryos with inner cells**
-	1 x EPI (3)	1 x full ICM , 1 x EPI (2)<br>1 x full ICM, 1 x PE (2)<br>2 x EPI (1)	3 x full ICM (1)<br>2 x full ICM, 1 x EPI (2)<br>1 x full ICM, 2 x EPI (2)<br>1 x full ICM, 2 x PE (1)<br>3 x EPI (1)	4 x full ICM (4)<br>3 x full ICM, 1 x EPI (2)<br>2 x full ICM, 2 x EPI (3)<br>1 x full ICM, 3 x EPI (2)

Supplementary Table S10. Developmental potential of sets of late quadruplets to generate inner cells. Abbreviations: EPI – an embryo that produced only EPI cells; PE – an embryo that generated only PE cells; ICM – an embryo that developed an ICM composed of two lineages: EPI and PE. The number of sets of late quadruplets with a given type of inner cell is given in parentheses.

**No inner cells**	**One embryo with inner cells**	**Two embryos with inner cells**	**Three embryos with inner cells**	**Four embryos with inner cells**
3	1 x PE (2)<br>1 x EPI (1)	2 x full ICM (2)<br>1 x full ICM, 1 x EPI (2)<br>1 x full ICM, 1 x PE (1)<br>2 x PE (2)<br>2 x EPI (1)	3 x full ICM (2)<br>2 x full ICM, 1 x PE (3)<br>2 x full ICM, 1 x EPI (1)<br>1 x full ICM, 2 x EPI (2)	4 x full ICM (3)<br>3 x full ICM, 1 x EPI (2)<br>3 x full ICM, 1 x PE (1)
